# Discovery of novel 1,2,3-triazole derivatives as anticancer agents using QSAR and in silico structural modification

**DOI:** 10.1186/s40064-015-1352-5

**Published:** 2015-10-05

**Authors:** Veda Prachayasittikul, Ratchanok Pingaew, Nuttapat Anuwongcharoen, Apilak Worachartcheewan, Chanin Nantasenamat, Supaluk Prachayasittikul, Somsak Ruchirawat, Virapong Prachayasittikul

**Affiliations:** Department of Clinical Microbiology and Applied Technology, Faculty of Medical Technology, Mahidol University, Bangkok, 10700 Thailand; Center of Data Mining and Biomedical Informatics, Faculty of Medical Technology, Mahidol University, Bangkok, 10700 Thailand; Department of Chemistry, Faculty of Science, Srinakharinwirot University, Bangkok, 10110 Thailand; Department of Clinical Chemistry, Faculty of Medical Technology, Mahidol University, Bangkok, 10700 Thailand; Laboratory of Medicinal Chemistry, Chulabhorn Research Institute, Bangkok, 10210 Thailand; Program in Chemical Biology, Chulabhorn Graduate Institute, Bangkok, 10210 Thailand; Center of Excellence On Environmental Health and Toxicology, Commission On Higher Education (CHE), Ministry of Education, Bangkok, Thailand

**Keywords:** Triazoles, Anticancer activity, Drug design, Computational chemistry, QSAR, Structural modification

## Abstract

Considerable attention has been given on the search for novel anticancer drugs with respect to the disease sequelae on human health and well-being. Triazole is considered to be an attractive scaffold possessing diverse biological activities. Structural modification on the privileged structures is noted as an effective strategy towards successful design and development of novel drugs. The quantitative structure–activity relationships (QSAR) is well-known as a powerful computational tool to facilitate the discovery of potential compounds. In this study, a series of thirty-two 1,2,3-triazole derivatives (**1**–**32**) together with their experimentally measured cytotoxic activities against four cancer cell lines i.e., HuCCA-1, HepG2, A549 and MOLT-3 were used for QSAR analysis. Four QSAR models were successfully constructed with acceptable predictive performance affording *R*_CV_ ranging from 0.5958 to 0.8957 and RMSE_CV_ ranging from 0.2070 to 0.4526. An additional set of 64 structurally modified triazole compounds (**1A**–**1R**, **2A**–**2R**, **7A**–**7R** and **8A**–**8R)** were constructed in silico and their predicted cytotoxic activities were obtained using the constructed QSAR models. The study suggested crucial moieties and certain properties essential for potent anticancer activity and highlighted a series of promising compounds (**21**, **28**, **32**, **1P**, **8G**, **8N** and **8Q**) for further development as novel triazole-based anticancer agents.

**Electronic supplementary material:**

The online version of this article (doi:10.1186/s40064-015-1352-5) contains supplementary material, which is available to authorized users.

## Background

Great attention has been given towards prevention and treatment of cancers with respect to the impact of disease sequelae on long term well-being of individuals (Vos et al. 2012). Cancers have been reported as one of the Global Burden of Diseases (World Health Organization 2008) and are estimated to be one of main causes of death in the coming decades (Mathers and Loncar 2006; Soerjomataram et al. 2012). Therefore, the search for novel anticancer agents has become one of prime interests in drug discovery and development.

1,2,3-Triazoles are nitrogen heterocycles capable of forming hydrogen bonds which improves their solubility and ability to interact with biomolecular targets (Vatmurge et al. 2008). The 1,2,3-triazoles are highly stable to metabolic degradation as compared to other compounds containing three adjacent nitrogen (N) atoms (Vatmurge et al. 2008). The triazoles have been used for broad therapeutic applications due to their diverse biological activities (Agalave et al. 2011) i.e., antimicrobial (Shivarama Holla et al. 1998; Prasad et al. 2009; Turan-Zitouni et al. 2005), antiviral (Masuda et al. 1975), antiinflammatory (Almasirad et al. 2004), analgesic (Almasirad et al. 2004), anticancer (Holla et al. 2002; Shivarama Holla et al. 2003; Pingaew et al. 2014a, b), antifungal (Manclús et al. 2008) and anticonvulsant (Amir and Shikha 2004) activities. In this regards, these privileged scaffolds have drawn considerable attention in the field of medicinal chemistry (Kumar and Kavitha 2013).

The computational approaches are widely known for their effectiveness in facilitating drug design and discovery (Kaul 1998). Quantitative structure–activity relationships (QSAR) is an in silico method for correlating structures of the compounds with their biological activities (Nantasenamat et al. 2009, 2010). QSAR can significantly reduce cost and time of drug discovery pipeline (Perkins et al. 2003) since the method provides beneficial knowledge for rational drug design such as crucial properties or moieties required for potent activities and pharmacokinetic information (Hansch et al. 2004). The QSAR models have been successfully constructed for understanding structure–activity relationships (SAR) of a wide range of bioactive compounds and diverse biological activities (Prachayasittikul et al. 2014; Nantasenamat et al. 2014; Worachartcheewan et al. 2012, 2013, 2014a, b, c).

Structural modification is extensively used to obtain potential lead compounds with improved potency and pharmacokinetic properties as well as reduced toxicities (Hughes et al. 2011; Anderson 2003; Prachayasittikul et al. 2014). The lack of structural diversity is one of current problems in the field of drug discovery which leads the growing awareness on expansion of chemical space (Barker et al. 2013; Dandapani and Marcaurelle 2010). The modification on privileged scaffolds is one of efficacious strategies to increase structural diversity thereby potentially addresses current issue. In addition, structural modification on the triazole pharmacophore has been noted as an efficient concept in the search for novel triazole drugs (Chrysina et al. 2009; Pingaew et al. 2014a, b).

**Fig. 1:**
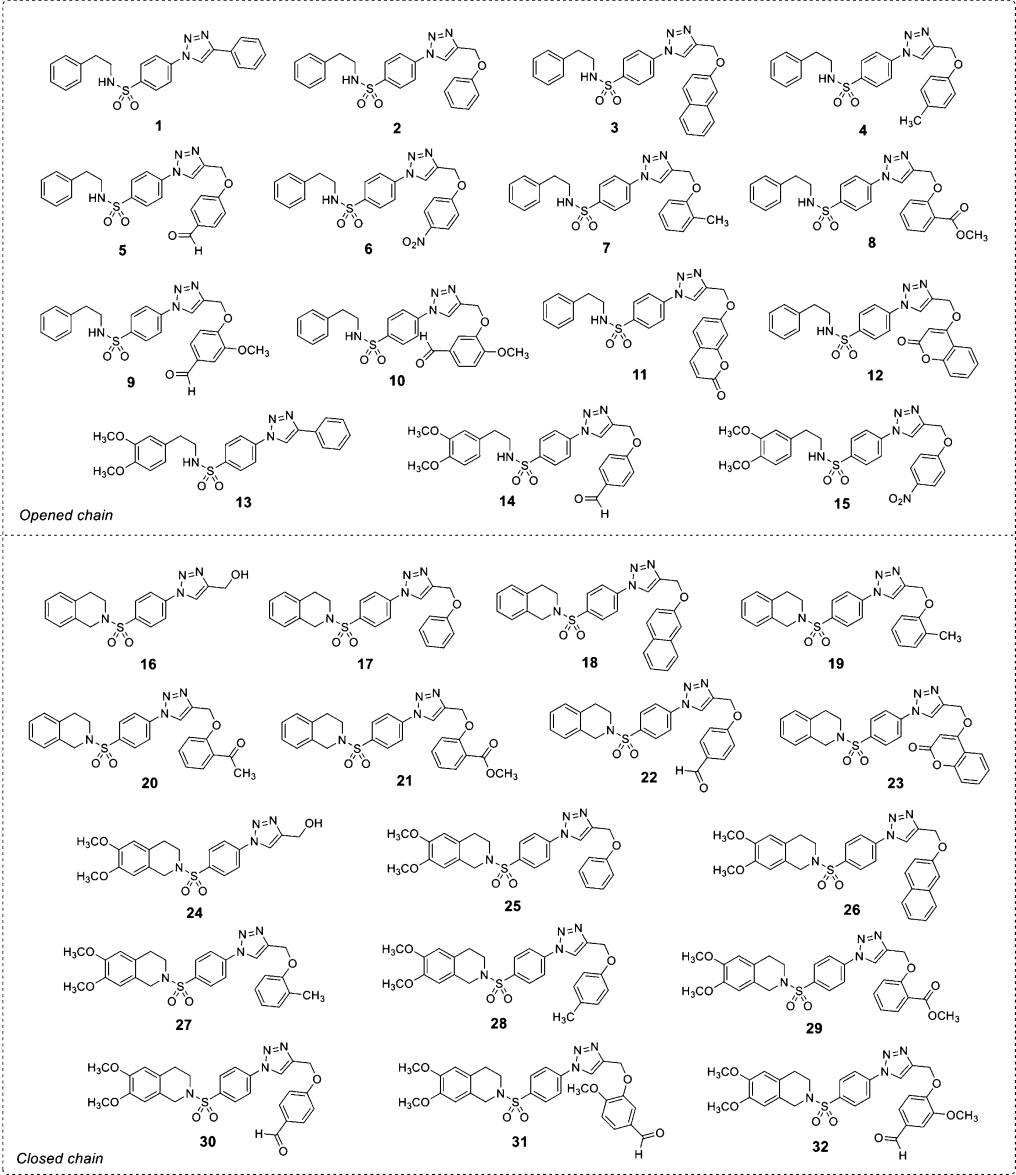
Chemical structures of tested compounds **1**–**32**

**Fig. 2:**
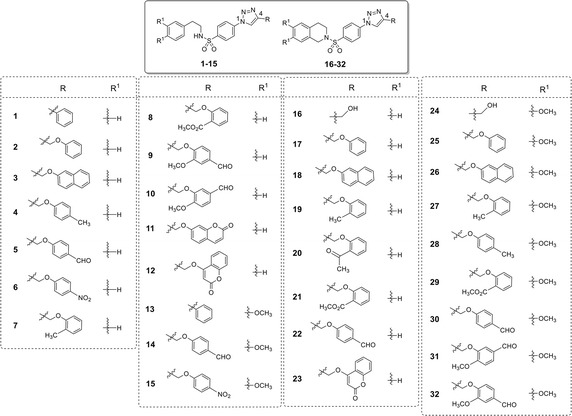
The substitutions on opened (**1**–**15**) and closed (**16**–**32**) chain core structures of 1,2,3-triazole

**Fig. 3:**
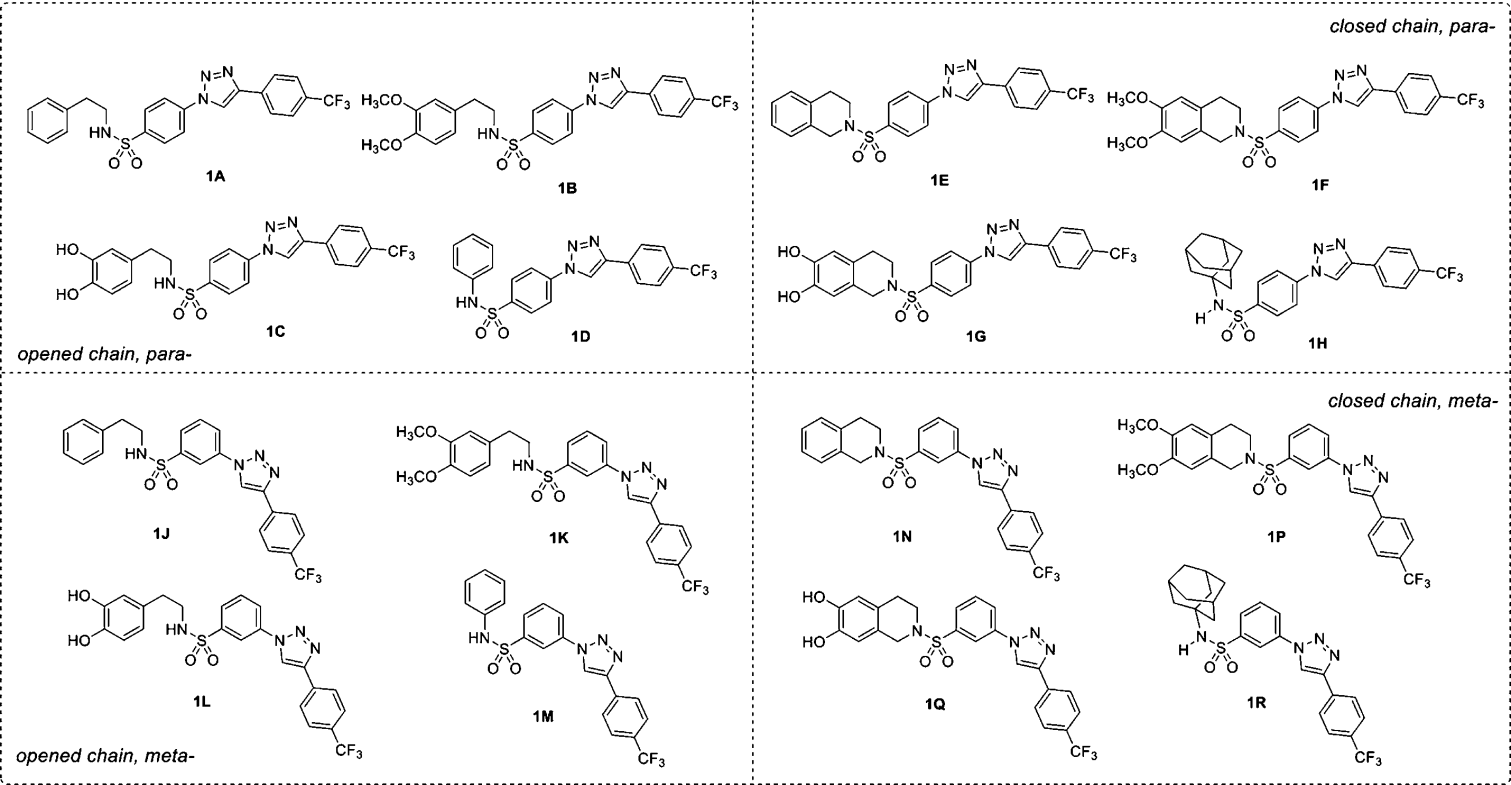
Chemical structures of modified compound series **1** (**1A**–**1R**)

**Fig. 4:**
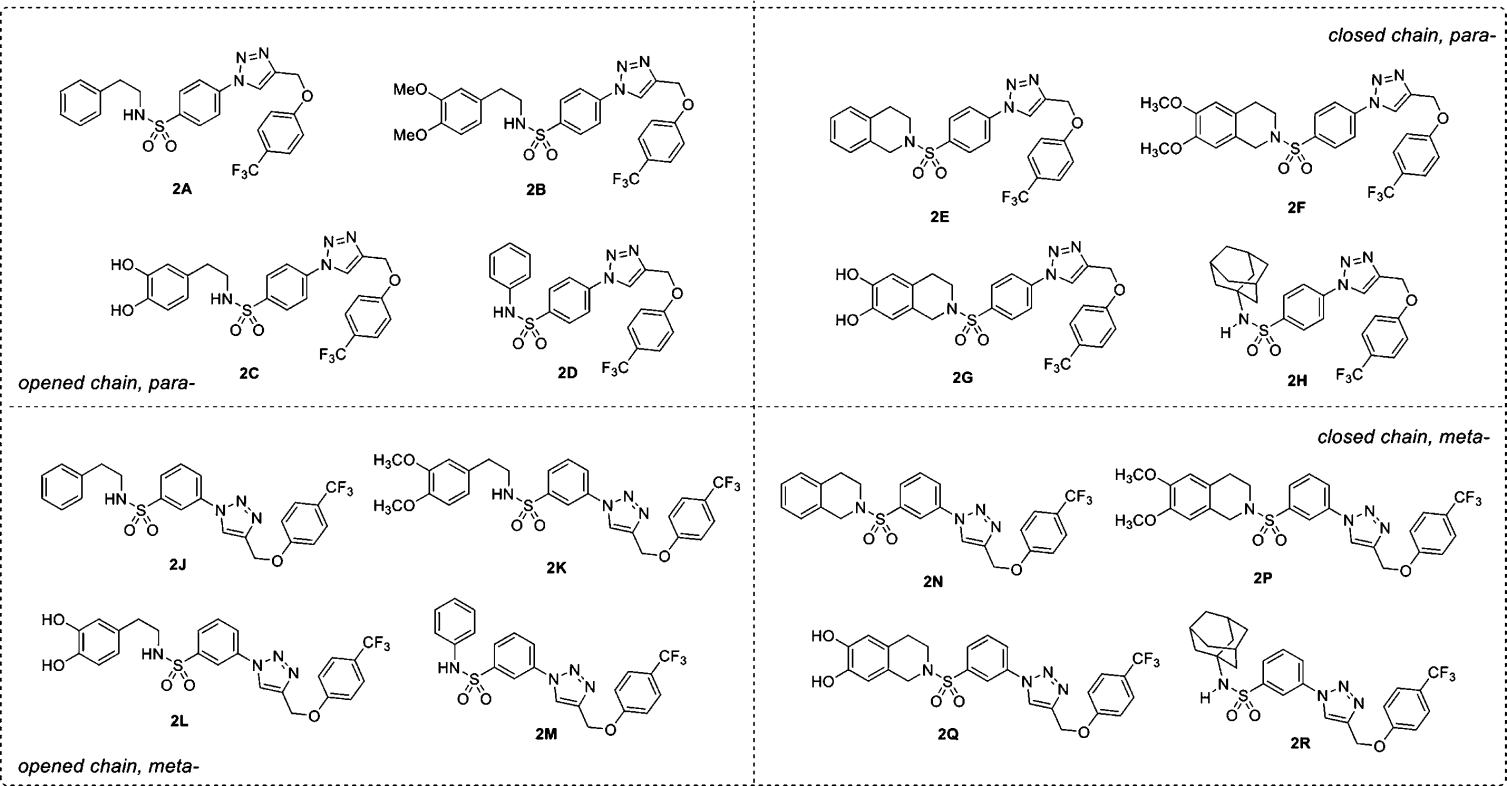
Chemical structures of modified compound series **2** (**2A**–**2R**)

**Fig. 5:**
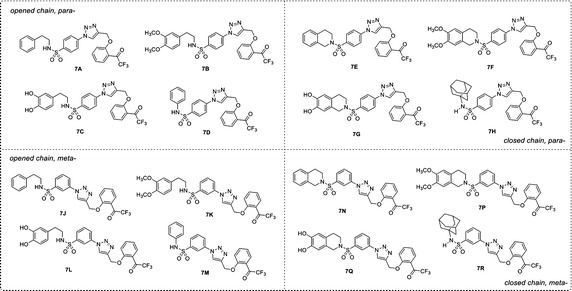
Chemical structures of modified compound series **7** (**7A**–**7R**)

**Fig. 6:**
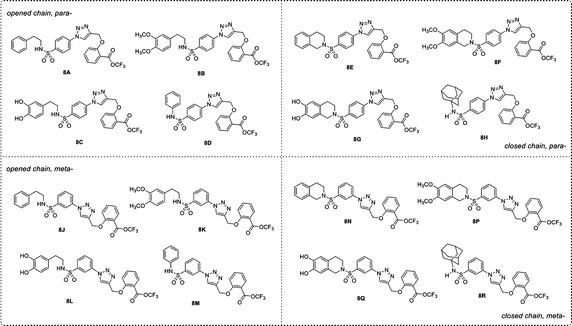
Chemical structures of modified compound series **8** (**8A**–**8R**)

**Fig. 7:**
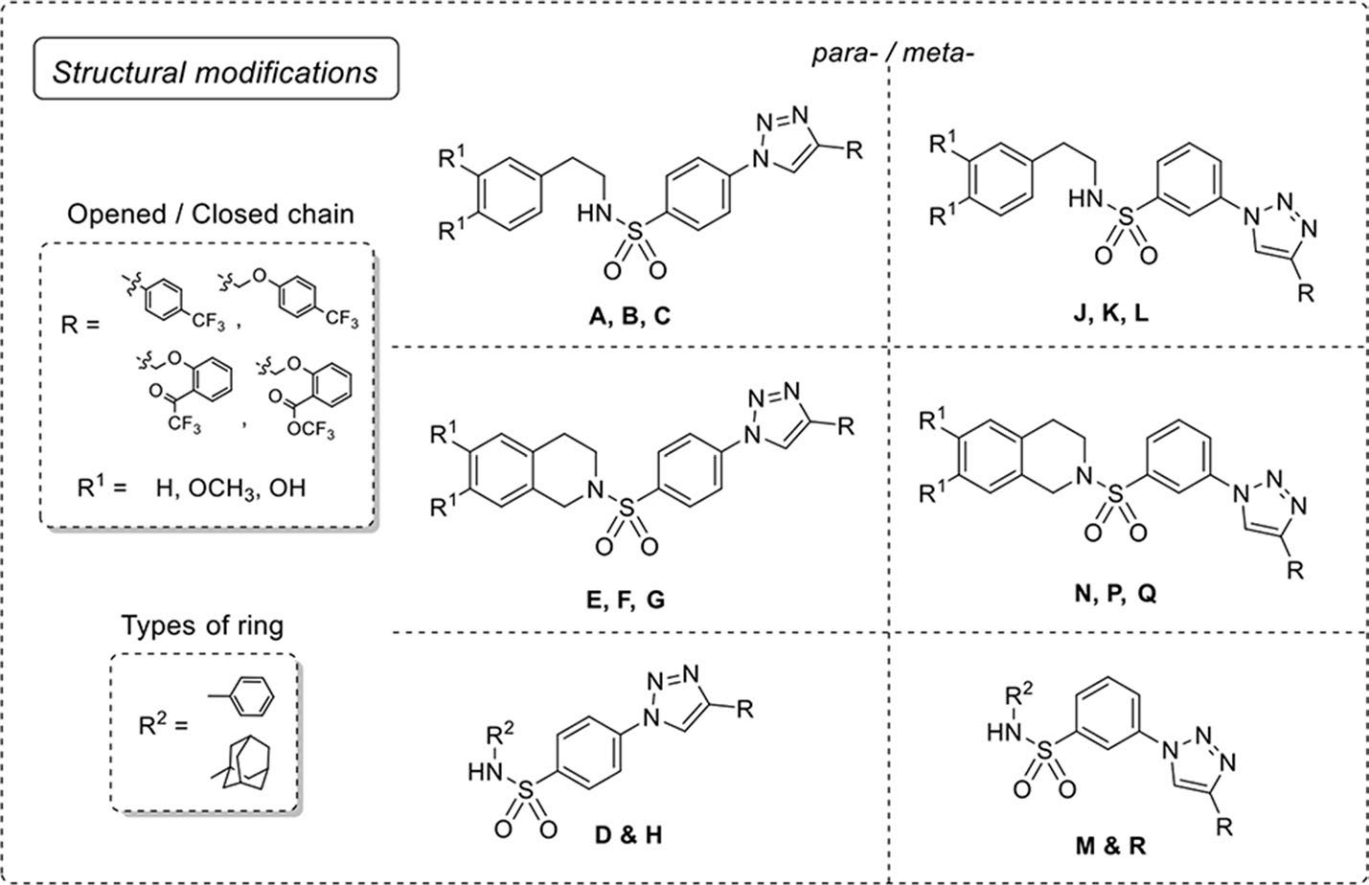
Modification on 1,2,3-triazole scaffold series **1**, **2**, **7** and **8**

Recently, a set of novel disubstituted 1,2,3-triazole derivatives (**1**–**32**, Fig. 1) has been reported as cytotoxic agents against four cancer cell lines i.e., HuCCA-1, HepG2, A549 and MOLT-3 by our research group (Pingaew et al. 2014a, b). Molecular docking of the tetrahydroisoquinoline-triazole derivatives **16**–**32** revealed that an aldo–keto reductase 1C3 (AKR1C3) has been identified to be a plausible target responsible for their anticancer activity (Pingaew et al. 2014a). In addition, the 1,2,3-triazoles (**2**–**7**, **12**–**13** and **15**) were shown to be aromatase inhibitors (Pingaew et al. 2015). The 1,2,3-triazole ring can be synthesized using copper catalyzed azide-alkyne cycloaddition (CuAAC), known as the Click reaction. The analytical data of the reported compounds is provided in supplementary data. These triazoles were substituted by phenylsulfonyl (opened and closed chain analogs containing substituent R^1^) at position 1; and by R group as phenyl and phenyl(naphthalenyl/coumaryl)oxymethyl at position 4. General core structures of compounds **1**–**32** are summarized as opened chain (**1**–**15**) and closed chain sulfonamides (**16**–**32**) as shown in Fig. 2. Triazole and sulfonyl moieties of compounds **1**–**32** are substituted at 1,4-positions (*para*-) of the phenyl ring. For simplification, compounds **1**–**32** will be denoted as *para*-trizoles. In this study, the QSAR was employed as a tool for understanding SAR of these 1,2,3-triazole derivatives. Four QSAR models were constructed using the chemical structure of the 32 tested compounds (**1**–**32)** along with their experimental cytotoxic activity. Furthermore, the application of constructed QSAR models were extended for the prediction of cytotoxic activity of an additional set of 64 structurally modified compounds (**1A**–**1R**, **2A**–**2R**, **7A**–**7R** and **8A**–**8R**, Figs. 3, 4, 5, 6) constructed in silico. Such structural modification of the compounds was rationally designed based on hydrophobic, electronic and steric effects as previously described by Topliss (1972, 1977). Therefore, the structurally modified compounds were obtained on the basis of changing groups on core structure (opened or closed chain), adding functional groups and altering the substitution positions of triazole and sulfonyl moieties on the phenyl ring (i.e., *para*- and *meta*-) to give *para*- and *meta*-triazoles, respectively (Fig. 7). A comprehensive analysis revealed important properties, crucial moieties and rigid analogs necessary for potent cytotoxic activity of the triazole compounds which would be of great benefit for guiding the design, screening and development of novel triazole anticancer drugs.

## Results and discussion

### Data sets

The data for QSAR analysis was obtained from the literature reported by our research group (Pingaew et al. 2014a, b). Analytical data of the reported compounds is provided in Additional file 1. The experimental cytotoxic activities (IC_50_) of the tested compounds (**1**–**32**) are shown in Table 1. The compounds (**1**–**32**) were classified by their IC_50_ values into four classes i.e., highly active (IC_50_ < 1 µM), moderately active (1 µM < IC_50_ < 10 µM), weakly active (IC_50_ > 10 µM) (Pérez-Sacau et al. 2007) and inactive (IC_50_ > 50 µg/mL) (Prachayasittikul et al. 2014). All inactive compounds were excluded from the QSAR analysis. Four QSAR models were separately constructed based on experimental testing against four cancer cell lines.

**Table 1:** Experimental cytotoxic activity of triazoles **1**–**32** against four cancer cell lines

Compound	IC_50_ (μM)			
	HuCCA-1	HepG2	A549	MOLT-3
**1**	8.65 ± 1.70^b^	9.07 ± 1.15^b^	34.54 ± 0.89^c^	Inactive^d^
**2**	Inactive^d^	57.54 ± 8.66^c^	Inactive^d^	Inactive^d^
**3**	Inactive^d^	28.21 ± 2.89^c^	Inactive^d^	74.23 ± 5.08^c^
**4**	Inactive^d^	81.75 ± 2.89^c^	Inactive^d^	Inactive^d^
**5**	87.89 ± 0.92^c^	100.54 ± 2.12^c^	Inactive^d^	32.02 ± 0.76^c^
**6**	Inactive^d^	Inactive^d^	Inactive^d^	61.42 ± 1.01^c^
**7**	Inactive^d^	41.62 ± 1.15^c^	Inactive^d^	34.24 ± 3.11^c^
**8**	Inactive^d^	49.40 ± 4.04^c^	Inactive^d^	8.81 ± 0.42^b^
**9**	Inactive^d^	57.52 ± 6.51^c^	79.18 ± 14.15^c^	9.22 ± 0.48^b^
**10**	Inactive^d^	34.51 ± 4.36^c^	39.04 ± 0.37^c^	10.33 ± 0.08^c^
**11**	16.12 ± 0.71^c^	12.44 ± 1.71^c^	19.60 ± 2.33^c^	88.97 ± 3.42^c^
**12**	Inactive^d^	Inactive^d^	Inactive^d^	10.65 ± 0.48^c^
**13**	Inactive^d^	23.89 ± 3.00^c^	18.19 ± 0.35^c^	60.99 ± 6.66^c^
**14**	Inactive^d^	Inactive^d^	28.03 ± 1.63^c^	17.43 ± 0.41^c^
**15**	Inactive^d^	Inactive^d^	Inactive^d^	10.10 ± 0.27^c^
**16**	51.35 ± 5.66^c^	Inactive^d^	Inactive^d^	Inactive^d^
**17**	Inactive^d^	6.50 ± 0.14^b^	Inactive^d^	Inactive^d^
**18**	Inactive^d^	60.48 ± 14.14^c^	Inactive^d^	Inactive^d^
**19**	Inactive^d^	Inactive^d^	66.30 ± 0.70^c^	Inactive^d^
**20**	30.16 ± 4.07^c^	19.12 ± 3.06^c^	14.90 ± 1.02^c^	21.86 ± 3.67^c^
**21**	0.63 ± 0.04^a,e^	12.36 ± 1.97^c^	0.57 ± 0.02^a,e^	18.63 ± 1.62^c^
**22**	Inactive^d^	5.27 ± 0.71^b^	59.07 ± 11.31^c^	Inactive^d^
**23**	24.80 ± 2.19^c^	Inactive^d^	25.29 ± 10.78^c^	80.78 ± 10.23^c^
**24**	72.0 ± 10.54^c^	31.79 ± 2.89^c^	41.04 ± 9.40^c^	Inactive^d^
**25**	Inactive^d^	2.57 ± 0.99^b^	Inactive^d^	Inactive^d^
**26**	Inactive^d^	1.26 ± 0.42^b^	Inactive^d^	36.35 ± 1.36^c^
**27**	39.71 ± 1.48^c^	1.48 ± 0.61^b^	27.21 ± 1.77^c^	Inactive^d^
**28**	inactive^d^	0.56 ± 0.01^a,e^	Inactive^d^	Inactive^d^
**29**	4.79 ± 0.28^b^	3.37 ± 0.96^b^	8.43 ± 2.79^b^	11.74 ± 4.97^c^
**30**	31.09 ± 8.91^c^	12.49 ± 2.47^c^	31.84 ± 8.13^c^	34.12 ± 0.97^c^
**31**	76.15 ± 1.77^c^	41.36 ± 2.89^c^	31.91 ± 9.76^c^	5.82 ± 0.85^b^
**32**	39.98 ± 4.03^c^	Inactive^d^	Inactive^d^	5.50 ± 0.61^b,e^
Etoposide^f^	–^g^	30.16 ± 0.50	–^g^	0.051 ± 0.002
Doxorubixin^f^	0.83 ± 0.07	0.79 ± 0.08	0.44 ± 0.01	–^g^

The compounds (**1**–**32**) were classified by their IC_50_ values into four classes i.e., highly active (IC_50_ < 1 µM), moderately active (1 µM < IC_50_ < 10 µM), weakly active (IC_50_ > 10 µM) (Pérez-Sacau et al. 2007) and inactive (IC_50_ > 50 µg/mL) (Prachayasittikul et al. 2014). All inactive compounds were excluded from the QSAR analysis. Four QSAR models were separately constructed based on experimental testing against four cancer cell lines

^a^Highly active compound

^b^Moderately active compound

^c^Weakly active compound

^d^Inactive compound

^e^The most potent compound against each cell line

^f^Reference drugs

^g^Not tested

### Obtaining informative descriptor values

Chemical structures of 32 triazole derivatives (**1**–**32**) were drawn, geometrically optimized, and calculated to obtain a set of descriptor values containing 13 quantum chemical descriptors and 3,224 molecular descriptors. The feature selection using correlation-based followed by stepwise multiple linear regression (MLR) methods was performed to select a set of significant informative descriptors of each cell line in which their definitions and values are shown in Table 2 and Additional file 2, respectively.

**Table 2:** Definition of descriptors using for development of QSAR models

Descriptor	Type	Definition
R5e+	GETAWAY descriptors	R maximal autocorrelation of lag 5/weighted by Sanderson electronegativity
nArCOOR	Functional group counts	Number of esters (aromatic)
RDF105m	RDF descriptors	Radial Distribution Function—105/weighted by mass
MATS7m	2D autocorrelations	Moran autocorrelation of lag 7 weighted by mass
MATS8v	2D autocorrelations	Moran autocorrelation of lag 8 weighted by van der Waals volume
Lop	lopping centric index	Topological indices
R7m	GETAWAY descriptors	R autocorrelation of lag 7/weighted by atomic masses

### QSAR analysis

**Fig. 8:**
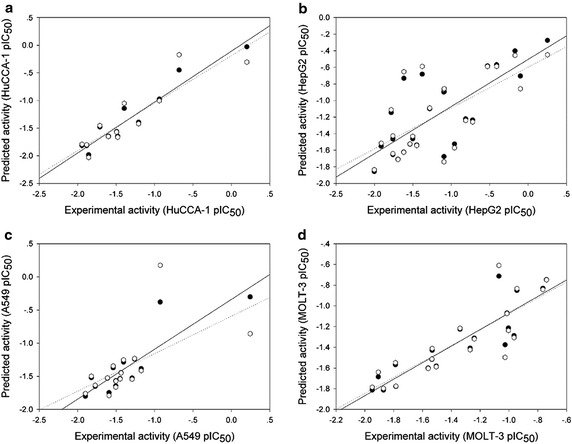
Plots of experimental versus predicted pIC_50_ values of cytotoxic activities against four cell lines (**a** HuCCA-1, **b** HepG2, **c** A549, **d** MOLT-3) generated by QSAR models (training set: compounds are represented by *closed circle* and regression *line* is shown as a *solid line*, leave-one-out validated testing set: compounds are represented by opened hex and regression line is shown as a *dotted line*)

The multiple linear regression (MLR) is one of the most popularly used machine learning algorithms for understanding SAR and it has been successfully employed for predicting bioactivities of diverse classes of compounds (Prachayasittikul et al. 2014; Worachartcheewan et al. 2012, 2013, 2014b, c). Regarding cytotoxic activity against four cancer cell lines, the data were separated into four data sets for QSAR analysis. Four QSAR models were successfully constructed by MLR method using a set of selected informative descriptor values and experimental cytotoxic activities (pIC_50_). The QSAR models and their predictive performance parameters are summarized in Table 3. Acceptable predictive performances were obtained from all constructed QSAR models with *R*_*cv*_ and RMSE_cv_ values ranging from 0.5958 to 0.8957 and 0.2070–0.4526, respectively. The highest performance was achieved from the HuCCA-1 model showing *R*_*cv*_ = 0.8957 and RMSE_cv_ = 0.2562 whereas the lowest performance was observed for A549 model (*R*_*cv*_ = 0.5958, RMSE_cv_ = 0.4211). The experimental and predicted cytotoxic activities against four cancer cell lines (pIC_50_) are shown in Table 4 and Fig. 8.

**Table 3:** Summary of QSAR models and their predictive performances against four cancer cell line

Cell line	Equation	N	*R*_Tr_	RMSE_Tr_	*R*_CV_	RMSE_CV_
*HuCCA*-*1*	*pIC*_*50*_ = −84.0157(*R5e*+) + 1.0288(*nArCOOR*) + 0.8738	13	0.9597	0.1603	0.8957	0.2562
*HepG2*	*pIC*_*50*_ = 0.0784(*RDF105* *m*) + 5.1878 (*MATS7* *m*) − 1.7524	24	0.7537	0.4006	0.6724	0.4526
*A549*	*pIC*_*50*_ = 1.5979(*MATS8v*) + 0.9251(*nARCOOR*) − 1.7829	16	0.8673	0.2390	0.5958	0.4211
*MOLT*-*3*	*pIC*_*50*_ = 1.0649(*Lop*) + 10.3977(*R7* *m*) − 5.6832	20	0.8936	0.1714	0.8430	0.2070

pIC_50_ is the concentration of compound required for 50 % inhibition of cell growth

*N* number of data set, *R*_*Tr*_ correlation coefficient of the training set, *RMSE*_*Tr*_ root mean square error of the training set, *R*_*CV*_ correlation coefficient of leave-one-out cross validation (LOO-CV) of the testing set, *RMSE*_*CV*_ root mean square error LOO-CV of the testing set

**Table 4:** Experimental and predicted cytotoxic activities (pIC_50_) of compounds **1**–**32** against cancer cell lines

Compound	HuCCA-1		HepG2		A549		MOLT-3	
	Exp.	Pred.	Exp.	Pred.	Exp.	Pred.	Exp.	Pred.
**1**	−0.937	−1.004	−0.958	−1.571	−1.538	−1.345	–^a^	–^a^
**2**	–^a^	–^a^	−1.760	−1.640	–^a^	–^a^	–^a^	–^a^
**3**	–^a^	–^a^	−1.450	−1.543	–^a^	–^a^	−1.871	−1.797
**4**	–^a^	–^a^	−1.912	−1.514	–^a^	–^a^	–^a^	–^a^
**5**	−1.944	−1.790	−2.002	−1.835	–^a^	–^a^	−1.505	−1.588
**6**	–^a^	–^a^	–^a^	–^a^	–^a^	–^a^	−1.788	−1.548
**7**	–^a^	–^a^	−1.619	−1.624	–^a^	–^a^	−1.535	−1.513
**8**	–^a^	–^a^	−1.694	−1.711	–^a^	–^a^	−0.945	−0.833
**9**	–^a^	–^a^	−1.760	−1.426	−1.899	−1.760	−0.965	−1.306
**10**	–^a^	–^a^	−1.538	−1.524	−1.592	−1.790	−1.014	−1.074
**11**	−1.207	−1.420	−1.095	−1.739	−1.292	−1.544	−1.949	−1.785
**12**	–^a^	–^a^	–^a^	–^a^	–^a^	–^a^	−1.027	−1.497
**13**	–^a^	–^a^	−1.378	−0.589	−1.260	−1.231	−1.785	−1.775
**14**	–^a^	–^a^	–^a^	–^a^	−1.448	−1.540	−1.241	−1.319
**15**	–^a^	–^a^	–^a^	–^a^	–^a^	–^a^	−1.004	−1.238
**16**	−1.711	−1.454	–^a^	–^a^	–^a^	–^a^	–^a^	–^a^
**17**	–^a^	–^a^	−0.813	−1.241	–^a^	–^a^	–^a^	–^a^
**18**	–^a^	–^a^	−1.782	−1.112	–^a^	–^a^	–^a^	–^a^
**19**	–^a^	–^a^	–^a^	–^a^	−1.822	−1.498	–^a^	–^a^
**20**	−1.479	−1.665	−1.281	−1.090	−1.173	−1.415	−1.340	−1.216
**21**	0.201	−0.306	−1.092	−0.858	0.244	−0.857	−1.270	−1.423
**22**	–^a^	–^a^	−0.722	−1.259	−1.771	−1.635	–^a^	–^a^
**23**	−1.394	−1.051	–^a^	–^a^	−1.403	−1.252	−1.907	−1.640
**24**	−1.857	−2.031	−1.502	−1.431	−1.613	−1.524	–^a^	–^a^
**25**	–^a^	–^a^	−0.410	−0.589	–^a^	–^a^	–^a^	–^a^
**26**	–^a^	–^a^	−0.100	−0.859	–^a^	–^a^	−1.561	−1.604
**27**	−1.599	−1.652	−0.170	−0.456	−1.435	−1.449	–^a^	–^a^
**28**	–^a^	–^a^	0.252	−0.449	–^a^	–^a^	–^a^	–^a^
**29**	−0.680	−0.173	−0.528	−0.590	−0.926	0.176	−1.070	−0.609
**30**	−1.493	−1.570	−1.097	−0.866	−1.503	−1.573	−1.533	−1.412
**31**	−1.882	−1.802	−1.617	−0.653	−1.504	−1.664	−0.765	−0.843
**32**	−1.602	−1.652	–^a^	–^a^	–^a^	–^a^	−0.740	−0.749

*Exp.* experimental activity, *Pred.* predicted activity

^a^Compounds determined to be experimentally inactive and were excluded from QSAR analysis

### Prediction of cytotoxic activities of virtually modified compounds

In order to investigate the effects of structural modification on the core structures of triazoles as opened chain (**1**–**15**) and closed chain (**16**–**32**), a series of structural modified compounds (**1A**–**1R**, **2A**–**2R**, **7A**–**7R** and **8A**–**8R**) were virtually constructed based on the changing substituents as shown in Fig. 7.

The modified compounds were drawn, geometrically optimized and calculated to obtain distinct sets of important descriptor values of each QSAR models (Additional file 2) for subsequently calculation of their predicted activities. The structurally modified compounds were categorized by their predicted cytotoxic activities as highly active (pIC_50_ > 0), moderately active (−1 < pIC_50_ < 0) and weakly to inactive (pIC_50_ < −1) (Prachayasittikul et al. 2014). The predicted cytotoxic activity (pIC_50_) of modified compound series are shown in Additional file 3.

### Understanding structure–activity relationships

Comprehensive consideration of experimental activities of the tested compounds (**1**–**32**) and predicted cytotoxic activities of modified compounds (**1A**–**1R**, **2A**–**2R**, **7A**–**7R** and **8A**–**8R**, Figs. 3, 4, 5, 6) against four cancer cell lines along with their descriptor values were performed to understand the SAR. The effects of structural modifications by changing substituents R and R^1^ (Figs. 2, 7) were observed and summarized in Additional file 4. The effects of substitutions on *meta*- and *para*-*trizoles* were compared based on the modified compound series obtaining the best improved activity against particular cancer cell line (Additional file 5).

#### HuCCA-1 cell line

1 $$pIC_{50}^{{}} = - 84.0157\left( {R5e + } \right) + 1.0288\;\left( {nArCOOR} \right) + 0.8738$$

The experimental results (Table 1) showed that the majority of the tested compounds (**1**–**32**) were weakly active or inactive against HuCCA-1 cell line except for ester compounds **21** (highly active), **1** (moderately active) and **29** (moderately active). Particularly, ester compound **21** was the most potent compound affording less IC_50_ value (0.63 µM) than the reference drug, doxorubicin (0.83 µM), followed by compound **29** as second ranked. The effect of opened/closed chain core structures were found in compounds **8** and **21** which are the compounds containing the same substituents (R) while bearing different core structures, opened chain (**8**) or closed chain (**21**), Fig. 2. Interestingly, the closed chain structure can shift the activity of the compound from inactive (**8**, opened chain) to highly active (**21**, closed chain). Moreover, compound **21** exhibited more potent activity than compound **29** (IC_50_ 4.79 µM) indicating that the replacement of H atoms (R^1^) in aromatic ring by methoxy (OCH_3_) groups can deteriorate the activity. The similar effects can be observed in compound **24** as compared to compound **16**. However, in some cases, the methoxy substituents (R^1^) can improve activity by changing inactive compounds to active compounds such as compound **19** (inactive) to **27** (active), and **22** (inactive) to **30** (active). Certain functional groups (R) were observed to influence the activity of particular compounds. It was found that replacement of naphthalenyl group in compound **3** (inactive) by 7-coumaryl group gives rise to improved activity of compound **11** (IC_50_ 16.12 µM) whereas 4-coumaryl substituent leads to inactive compound **12**. In addition, the effect of the closed chain was noted to improve activity of 4-coumarin analogs as found in compound **23** (IC_50_ 24.80 µM) when compared to opened chain compound **12** (inactive).

The QSAR analysis revealed that descriptors pertaining to the electronegativity (R5e+) and the number of aromatic esters (nArCOOR) of the compounds influenced the cytotoxic activity against HuCCA-1 cell line, Eq. 1. The R5e+ descriptor was the most influential descriptor as represented by its high regression coefficient. The negative regression coefficient of R5e+ (−84.0157) and positive regression coefficient of nArCOOR (1.0288) indicate that low electronegativity and high number of aromatic esters are required for preferable cytotoxic activity. It was observed that compounds with high value of R5e+ exhibited poor activity. For example, ketone **7D** (ArCOCF_3_, Fig. 5) with high electronegativity atom (F), which had the highest value of R5e+ (0.057, Additional file 2) amongst all modified compounds, exhibited the worst activity with pIC_50_ value of −3.915 (Additional file 3). The presence of aromatic esters was found to be important for potent activity as seen in compounds **21**, **29** and modified compounds (ArCOOCF_3_) in series **8** (**8A**–**8R**) as shown in Fig. 6.

Considering the predicted cytotoxic activity of modified compounds (Additional file 3) and experimental activity of their prototypes (Table 4), the structural modification of compound **1** reduce cytotoxic activity. The experimental inactive compounds (**2** and **7**) afforded modified compounds (**2A**–**2R** and **7A**–**7R**) that possessed weakly to inactive cytotoxic activity. Although the tested compound **8** contains aromatic ester, which is represented by nArCOOR descriptor value, it was found to be experimentally inactive. However, the improved activities of modified compounds in series **8** (**8A**–**8R**) were predicted when compared to their inactive prototype (**8**). Notably, the ester **8N** (pIC_50_ 0.054) was noted as the most potent compound among all modified compound series. It was observed that particular forms (opened/closed chain), substituents R (phenoxyester), R^1^ (H, OH, OCH_3_) and R^2^ (1-adamantyl, phenyl) on the *meta*- and *para*- triazoles influence the R5e+ value of the compounds in governing their cytotoxic activities. For both *para*- and *meta*- modified compounds in series **8**, closed chain analogs (**8E**, **8G**, **8N** and **8P**) were predicted to exhibit more potent cytotoxic activity (indicated by pIC_50_ value) than opened chain analogs (**8A**, **8C**, **8J** and, **8K**) and the 1-adamantyl (R^2^) derivatives (**8H** and **8R**) were predicted to be more active than phenyl (R^2^) derivatives (**8D** and **8M**). In case of the opened chain analogs (**8A**, **8B**, **8C**, **8J**, **8K** and **8L**), most of the compounds with *para*-triazole were predicted as more potent compounds than the compounds with *meta*-triazole as indicated by pIC_50_ values i.e., **8A** (−0.618) > **8J** (−1.038) and **8B** (−0.786) > **8K** (−1.122) except for the case of phenyl (R^2^) compounds in which the large improved activity of *meta*-triazole **8M** (−0.618) was observed as compared to the *para*-triazole **8D** (−2.466). In contrast, the closed chain *meta*-triazole compounds (**8N** and **8P**) and 1-adamantyl *meta*-triazole compound (**8R**, pIC_50_ −0.282) were found to exhibit more potent activity than the corresponding closed chain *para*-triazoles (**8E** and **8F**, pIC_50_ −0.114 and −0.786, respectively) and 1-adamantyl (R^2^) *para*-triazole compound (**8H**, pIC_50_ −0.702).

The most potent ester (COOCF_3_) **8N** is the closed chain *meta*-triazole (R^1^=H), which had the lowest R5e+ value (0.022) among the modified compounds. The most potent *para*-triazole methyl ester (COOCH_3_) **21** (R^1^=H) had lower value of R5e+ (0.023) than its methoxy analog **29** (R^1^ = OCH_3_, R5e+ = 0.028), thereby giving rise to higher potency of **21**. In contrast, the opened chain *para*-triazole ester (COOCH_3_) **8** (R^1^=H) exhibited no cytotoxic activity. This could be possibly explained that the cytotoxic activity against HuCCA-1 cell required the molecule with low electronegativity (R5e+) and less flexibility or more rigidity in interacting with the target site of action as observed in compound **21** comparing to its opened chain analog **8** (inactive compound).

#### HepG2 cell line:

2 $$pIC_{50}^{{}} = 0.0784\;\left( {RDF105m} \right) + 5.1878\;\left( {MATS7m} \right) + 1.7524$$

Results showed that most of the tested compounds (**1**–**32**) exhibited more potent cytotoxic activity against HepG2 cell lines than the reference drug, etoposide (Table 1). The closed chain analogs (**17**, **20**–**22** and **25**–**30**) displayed higher activity as compared to the opened chain analogs (**1**, **3**, **11** and **13**). Particularly, compound **28** was noted as the best compound affording less IC_50_ value (0.56 µM) than both reference drugs, etoposide (30.16 µM) and doxorubicin (0.79 µM). It was found that introducing the NO_2_ and 4-coumaryl moieties into the phenoxy ring of compound **2** leads to inactive cytotoxic activity of compounds **6** and **12**, respectively. It was observed that the substitution of OCH_3_ (R^1^) on aromatic ring increases cytotoxic activity of the compounds except for compounds **13**, **14**, and **30**. In contrast to HuCCA-1 cell line, the substitution of OCH_3_ on the aromatic ring (R^1^) can markedly increase the value of MATS7 giving rise to an improved activity of compound **29** (MATS7 = 0.040, Additional file 2 and IC_50_ = 3.37, Table 1) when compared to non-substituted (R^1^=H) compound **21** (MATS7 = −0.020, Additional file 2 and IC_50_ = 12.36, Table 1) as represented by approximately fourfolds reduced IC_50_ value. Among the experimental tested compounds, the closed chain derivatives exhibited more potent activity than that of the opened chains, except for closed chain compound **19** (inactive) comparing to its opened chain analog **7** (active). In addition, it should be noted that the *para*-position of CH_3_ on the phenoxy ring (**28**) has greater influence on enhancing the cytotoxic activity of the compound than the *ortho*-position (**27**).

The QSAR analysis revealed that mass descriptors i.e., RDF105m and MATS7m, are important descriptors for cytotoxic activity against HepG2 cell line, Eq. 2. The higher regression coefficient of MATS7m (5.1878) indicated its greater influential effect. The QSAR equation indicated that high values of RDF105m and MATS7m are essential for potent cytotoxic activity. For example, the highest RDF value (14.609) together with high MATS7m value (0.064) were found in the most potent compound (**28**) in the tested series, Additional file 2. Similarly, the most potent predicted cytotoxic activity of the modified compounds was noted in compound **1P** which had the high RDF values of 18.976.

Improved cytotoxic activities were predicted from all series of the modified compounds in which the most enhanced effects were noted for the modified compounds in series **1** and **7**. Compound **1P** was predicted as the most potent compound of the modified series followed by compound **7F** affording pIC_50_ values of −0.151 and −0.163, respectively (Additional file 3). Considering the modified series **1** and **7** (Figs. 3, 5, 7), the 1-adamantyl (R^2^) substituted (**H** and **R**) and closed chain (**E**, **F**, **G**, **N**, **P** and **Q**) compounds are more potent cytotoxic agents against HepG2 cell line than the phenyl (R^2^) substituted (**D** and **M**) and opened chain (**A**, **B**, **C**, **J**, **K** and **L**) compounds except for the compounds in series **7** (**7N** and **7P**). The better predicted activities were observed in the opened chain analogs i.e., **7J** and **7K** comparing to the closed chain analogs **7N** and **7P** (Additional file 3). For the opened chain in series **1** (**A**, **B**, **C**, **J**, **K** and **L**), phenyl (R^2^) substituted compounds (**D** and **M**) and 1-adamantyl (R^2^) substituted compounds (**H** and **R**), it was found that the *meta*-triazoles (**J**, **K**, **L**, **M** and **R**) exhibited better activities than the *para*-triazoles (**A**, **B**, **C**, **D** and **H**). For closed chain compounds (**E**, **F**, **G**, **N**, **P** and **Q**), the *meta*-triazole of series **1** (**1N**, **1P** and **1Q**) exerted more potent activity than *para*-triazole (**1E**, **1F** and **1G**). In contrast, the *para*-triazole of series 7 (**7E**, **7F** and **7G**) exhibited better activity than the *meta*-triazole (**7N**, **7P** and **7Q**). Particularly, compound **7F** afforded the predicted pIC_50_ value approximately tenfolds greater than **7P** (**7F**: pIC_50_ = −0.163, **7P**: pIC_50_ = −1.177, Additional file 3). In addition, the effects of *para*-triazole with closed chain and 1-adamantyl substituent (R^2^) on improving cytotoxic activity were generally observed via increased MATS7m values when compared to opened chain and phenyl (R^2^) substituted triazoles whereas that of *meta*-triazoles were governed by increased RDF105m values when compared to *para*-triazoles (Additional file 2).

Cytotoxic activity of *meta*- and *para*-triazoles are governed by substituents R, R^1^ and R^2^ in providing high values of descriptors (MATS7m and RDF105m) weighted by mass. Obviously, the most potent compounds **28** and **1P** constitute the same methoxy group (R^1^=OCH_3_), and R=*para*-methylphenoxymethyl and *para*-trifluoromethylphenoxy for **28** and **1P**, respectively. Such substituents (R and R^1^) enhanced masses on to the triazole core structures.

#### A549 cell line

3 $$pIC_{50}^{{}} = 1.5979\;\left( {MATS8v} \right) + 0.9251\;\left( {nArCOOR} \right) - 1.7829$$

Similar to the HuCCA-1 cell line, the majority of the tested compounds (**1**–**32**) exhibited weakly active and inactive cytotoxic activities against A549 cell line (Table 1). However, the ester compound **21** (highly active) was noted as the most potent cytotoxic agent followed by ester compound **29** (moderately active). It was found that closing the ring caused dramatically changes in activity shifting from inactive to active compounds. The notable effect was observed when compared the opened chain ester **8** (inactive) and closed chain ester **21** (highly active). Furthermore, the relative position of OCH_3_ and CHO on the substituted phenoxy ring was noted for its influence on cytotoxic activity in which these functional groups were suggested to be placed in *para*-position to each other as observed for compound **31** (active) rather than in *meta*-position as seen in compound **32** (inactive).

The QSAR analysis revealed that van der Waals volume descriptor (MATS8v) and the number of aromatic esters (nArCOOR) are important descriptors for cytotoxic activity against A549 cell line, Eq. 3. The positive regression coefficients of both descriptors indicated that high values are essential for potent cytotoxic activity of the compounds. The effects of substituents (R, R^1^) in the tested compound series (**1**–**32**) were observed via an alteration of MATS8v values. For example, the insertion of carbonyl group to the CH_3_ group of compound **19** (MATS8v = 0.164, Additional file 2 and IC_50_ 66.30 µM, Table 1) provided the ketone compound **20** (IC_50_ 14.90 µM) with approximately twofold increased MATS8v value (0.249, Additional file 2) giving rise to its improved activity when compared to compound **19**. Interestingly, replacement of CH_3_ group of compound **19** by COOCH_3_ gave the most potent compound **21** with the highest MATS8v (0.348) amongst the tested compounds (**1**–**32**). The potency of these compounds (**19**–**21**) are shown to be **21** > **20** > **19** with the MATS8v values of 0.348, 0.249 and 0.164, respectively. Similar findings of more active compounds possessing the higher MATS8v values were found, for example, compounds **10** > **9** and **27** > **28** (Additional file 4).

The structurally modified compounds in series **1** and **8** were predicted to be more potent compounds than their prototypes. Notably, all of the modified compounds in series **8** were predicted as moderately active compounds (Additional file 3) which may be governed by the presence of the aromatic ester in the molecule, as represented by the nArCOOR values (Additional file 2). Ester compounds **8G** and **8B** were noted as the most (MATS8v = 0.150) and the second most (MATS8v = 0.146) potent compounds among all modified compounds, respectively (Additional file 3). Apparently, both opened and closed chain *para*-triazole analogs of the modified compounds in series **8** exhibited more potent activities than their *meta*-triazole analogs (Additional file 5). The closed chain compounds (**E**, **G** and **N**, Fig. 6) were predicted to be more active than the opened chain compounds (**A**, **C** and **J**, Fig. 6) except for the opened chain analogs **8B** and **8K** were predicted as more potent compounds than the closed chain derivatives **8F** and **8P**, Additional file 4. However, effect of opened/closed chain were not clearly observed for OH (R^1^) substituted compounds **8L** (opened chain, pIC_50_ = −0.779, Additional file 3) and **8Q** (closed chain, pIC_50_ = −0.778, Additional file 3) as represented by their comparable pIC_50_ values. For both *para*- and *meta*- analogs of all modified series (Fig. 6), 1-adamantyl (R^2^) substituted compounds exhibited more potent activity than phenyl (R^2^) substituted compounds, except for series **1**. The phenyl (R^2^) substituted compound of *meta*-series, compound **1M**, was more potent than its 1-adamantyl (R^2^) analog **1R** (Additional file 4). No effect of changing substituted ring type (R^2^) was found for *para*-series as observed from comparable cytotoxic activities of compounds **1H** and **1D**, Additional file 4.

The most potent esters **21** (COOCH_3_, R^1^=H) and **8G** (COOCF_3_, R^1^=OH) are *para*-triazoles, in which both compounds had the highest values of MATS8v in their tested and modified series compounds, respectively.

#### MOLT-3 cell line

4 $$pIC_{50}^{{}} = 1.0649\left( {Lop} \right) + 10.3977\left( {R7m} \right) - 5.6832$$

Among the tested compounds (**1**–**32**), only compounds **8**, **9**, **31** and **32** were noted as moderately active cytotoxic agents against MOLT-3 cell line while the rest were listed as inactive to weakly active compounds. The findings suggested that the substitution of CO_2_CH_3_, OCH_3_ and CHO moieties on the phenoxylmethyl group (R) may essential for cytotoxic activity of the compounds. Interestingly, the effects of certain moieties and isomeric forms on cytotoxic activity against the MOLT-3 cell line were found in contrast to other tested cell lines i.e., HuCCA-1, HepG2 and A549. Particularly, *m*-substitution of OCH_3_ and CHO on phenoxy (R) group exerted the most potent activity as noted for closed chain compound **32** comparing to *p*-substitution of compound **31** (Additional file 4). Similar isomeric effect of OCH_3_ and CHO was found in opened chain analogs of *meta*- (**9**) and *para*- (**10**) in which **9** exhibited more potent activity than **10**. In addition, the replacement of naphthalenyl moiety in the opened chain triazoles (**3**) by 7-coumaryl moiety gave compound **11** with loss of the cytotoxic activity (**3** **>** **11**). However, more potent activity can be obtained by substitution of 4-coumaryl rather than 7-coumaryl (**12** > **11**), Table 1. Furthermore, opened chain triazole of 4-coumaryl (**12**) exhibited better activity when compared to the same substitution (R) on the closed chain triazole (**23**).

The QSAR analysis indicated that topological index (Lop) and atomic masses descriptor (R7m) were influential descriptors for affecting the cytotoxic activity against MOLT-3 cell line, Eq. 4. Regarding the regression coefficient of R7m, the importance of the atomic masses of the compounds were noted. The effect of functional group substitution affecting the R7 m values were obviously seen in closed chain (R^1^=OMe) compounds **31** and **32** in which both compounds possess the same value of Lop (0.876, Additional file 2) but different values of R7m (**31** = 0.377, **32** = 0.385, Additional file 2). It was observed that the relative positions of OCH_3_ and CHO on the phenoxylmethyl ring (R) affects the R7 m values. The higher value of R7m was observed when these two functional groups are placed in *meta*-position to each other thereby giving rise to more potent activity (**32** > **31**, Fig. 2, Additional file 3). Similar substitution effects of OCH_3_ and CHO on the R group afforded the same Lop value (0.740) were noted for opened chain (R^1^=H) compounds **9** and **10**. However, *meta*-substitution of OCH_3_ and CHO (**9**) displayed higher activity with relative lower R7m value as compared to *para*-substitution on the R group of **10**.

It was found that the structural modifications can improve the activities of all modified compounds (Table 4 and Additional file 3) when compared to their prototypes (**1**, **2**, **7** and **8**) except for 1-adamantyl analog (R^2^) of compound **7**, **7H** affording pIC_50_ value of −1.865 (Additional file 3) while that of **7** was −1.513 (Table 4). The best enhancing effects were found in modified compounds series **8** in which most of the compounds were predicted as highly active (Additional file 3). Interestingly, closed chain (R^1^=OH, R=*ortho*-ester) of *m*-triazole compound **8Q** (R7m = 0.537) and of *p*-triazole **8G** (R7m = 0.519) had the same value of Lop (0.983), and were found to be the most and the second most potent compounds of all modified series, respectively. However, none of them exhibited more potent activity than the reference drug, etoposide. Considering the modified compounds series **8**, the closed chain triazole derivatives (**8F**, **8G**, **8P** and **8Q**) exhibited more potent cytotoxic activity than the opened chain analogs except for **8A** and **8J** (**8A** > **8E** and **8J** > **8N**). The notably enhanced effect was observed in the closed chain hydroxyl (R^1^) derivative **8Q** as represented by its 8.61-folds pIC_50_ value (0.947) greater than the opened chain compound **8L** (0.110), Additional file 3.

A variety of structural modification effects on cytotoxic activity against the MOLT-3 cell line can be observed through *para*- and *meta*-triazoles (Additional file 5). It was noted that both *meta*- and *para*-triazoles influence the cytotoxic activity via the alteration of R7m values in which the more active compounds possess the higher values of R7m but equal Lop as compared to the less active ones (Additional files 2, 3). For example, *meta*-triazole compound **8M** (R^2^=phenyl) with higher R7m value was approximately twofold more potent than *para*-triazole of phenyl (R^2^) compound **8D** (R7m; **8M** = 0.397, **8D** = 0.365, Additional file 2 and pIC_50_; **8M** = −0.485, **8D** = −0.818, Additional file 3). The 1-adamantyl (R^2^) substitution in *para*-triazole core structure enhances cytotoxic activity as represented by 2.456 folds increased pIC_50_ value of compound **8H** (pIC_50_ = −0.333, Additional file 3) as compared to the phenyl (R^2^) substituted *para*-compound **8D** (pIC_50_ = −0.818, Additional file 3). In contrast, the phenyl (R^2^) substituted *meta*-triazole compound **8M** exhibited better activity than the 1-adamantyl (R^2^) substituted *meta*-triazole compound **8R** (pIC_50_: **8M** = −0.485, **8R** = −0.645, Additional file 3).

The most potent compounds (**32** and **8Q**) against MOLT-3 cell are *para*-triazole (R^1^=OCH_3_, R=phenoxyaldehyde) and *meta*-triazole (R^1^=OH, R=phenoxyester), respectively. It is notable that a carbonyl (CO) group of the aldehyde **32** may act as electrophilic center in interacting with nucleophile at the target site of action. Similar interaction could possibly be seen in the CO group of ester **8Q** with nucleophilic site of action. These findings suggested that particular isomers in combination with certain functional groups (R^2^) are required for preferable cytotoxic activity of the compounds. The promising compounds to be further developed as anticancer agents against each cancer cell line are summarized in Table 5, obviously, *meta*- triazoles **8N**, **1P** and **8Q** for HuCCA-1, HepG2 and MOLT-3 cell lines, respectively, and *para*- triazole **8G** for A549 cell line.

**Table 5:**
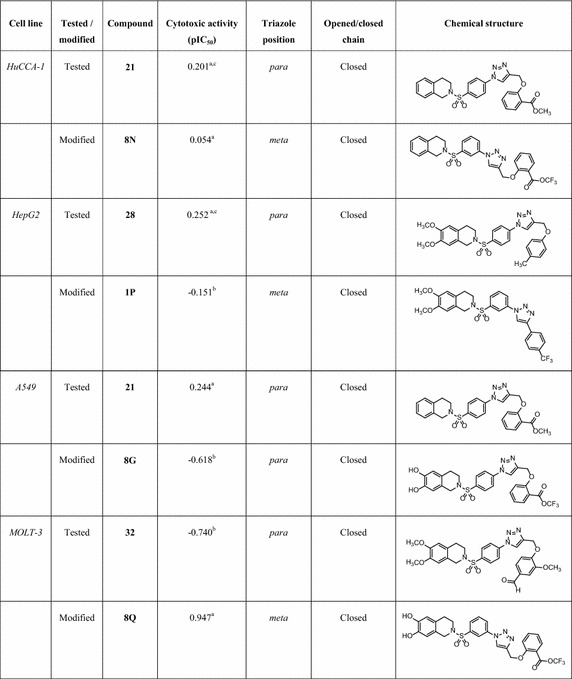
A summary of potential compounds for further development

^a^Highly active

^b^Moderately active

^c^Potent than reference drug

## Conclusion

Understanding structure–activity relationships of the compounds is essential for efficacious design and development of drugs. Herein, the QSAR was used as a tool for investigating the effects of structural modifications on the cytotoxic activity of the triazole derivatives. Four QSAR models were successfully constructed using the chemical structures and the experimental cytotoxic activities of a set of triazole derivatives (**1**–**32**). The acceptable predictive performances were obtained affording *R*_CV_ values ranging from 0.5958 to 0.8957 and RMSE_CV_ ranging from 0.2070 to 0.4526. The QSAR models revealed a set of important descriptors that represented distinct properties influencing the cytotoxic activity against particular cell lines i.e., electronegativity, number of aromatic ester, van der Waals volume, topological indice and atomic masses. The effects of *meta*-/*para*-triazoles, the opened/closed chain core structure and particular substituents (R, R^1^, R^2^) affecting the cytotoxic activities of the compounds were investigated by virtual construction of an additional set of structurally modified compounds (**1A**–**1R**, **2A**–**2R**, **7A**–**7R** and **8A**–**8R**) in which their cytotoxic activities were predicted using the constructed QSAR models obtained from the tested compounds. The findings indicated a set of potential compounds for further development as summarized in Table 5. The study provided insights into the SAR of the triazole derivatives and their cytotoxic activities. The influential moieties and isomers for preferable activity, and a set of promising compounds for further development were indicated for the benefit of guiding the design, synthesis and development of novel triazole-based anticancer agents.

## Methods

**Fig. 9:**
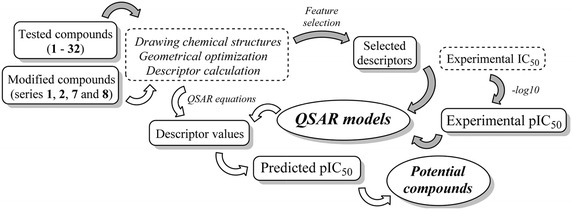
Workflow of the study

Conceptually, a series of predictive QSAR models were constructed using data sets obtained from experimentally tested compounds (**1**–**32**, Figs. 1, 2) (Pingaew et al. 2014a, b) and were subsequently used for prediction of additional structurally modified compounds (**1A**–**1R**, **2A**–**2R**, **7A**–**7R** and **8A**–**8R**, Fig. 3, 4, 5, 6) generated in silico. The conceptual framework is shown in Fig. 9. Initially, the molecular structures of the tested compounds (**1**–**32**) were drawn, geometrically optimized and calculated to obtain a large set of descriptor values. Hence, feature selection was performed to select only informative ones which were further used as predictors (*X*) to predict anticancer activity (*Y*). These *X* (descriptors) and *Y* (cytotoxic activity) blocks of data were subsequently used for development of QSAR models. Likewise, the construction, geometrical optimization and calculation were performed in the same manner with the structurally modified compounds (**1A**–**1R**, **2A**–**2R**, **7A**–**7R** and **8A**–**8R**) to obtain their descriptor values. Finally, the QSAR equations constructed by the tested compounds (**1**–**32**) were used to calculate predicted activities of modified compounds (**1A**–**1R**, **2A**–**2R**, **7A**–**7R** and **8A**–**8R**).

### Data sets

A set of triazole derivatives (**1**–**32**) and their experimental IC_50_ values against four cancer cell lines i.e., HuCCA-1, HepG2, A549 and MOLT-3 (Table 1) were obtained from literature reported by our research group (Pingaew et al. 2014a, b). In order to obtain normal distribution of data points, the IC_50_ values were converted to pIC_50_ values by taking the negative logarithm to the base of 10 (−log IC_50_). With respect to the anticancer activity against four cancer cell lines, four data sets were arranged for the construction of separated QSAR models. For each data set, the compounds exhibited inactive cytotoxic activity were excluded from the analysis.

### Molecular structure optimization and descriptor calculation

The rationale for geometrical optimization is to obtain low-energy conformers for investigated compounds that will subsequently be used for calculation of molecular descriptors. Chemical structures of the 32 tested compounds (**1**–**32**) and 64 virtually modified compounds (**1A**–**1R**, **2A**–**2R**, **7A**–**7R** and **8A**–**8R**) were drawn by the GaussView software (Dennington et al. 2003) and initially geometrically optimized using Gaussian 09 (Frisch et al. 2009) at the semi-empirical Austin Model 1 (AM1) level followed by density functional theory (DFT) calculation using the Becke’s three-parameter hybrid method with the Lee–Yang–Parr correlation functional (B3LYP) together with the 6–31 g(d) level. A set of quantum chemical descriptors consisted of the mean absolute atomic charge (*Q*_m_), total energy (*E*_total_), total dipole moment (*μ*), highest occupied molecular orbital energy (*E*_HOMO_), lowest unoccupied molecular orbital energy (*E*_LUMO_), energy difference of HOMO and LUMO (HOMO-LUMO_Gap_), electron affinity (EA), ionization potential (IP), Mulliken electronegativity (*χ*), hardness (*η*), softness (*S*), electrophilic index (*ω*_i_) and electrophilicity (*ω*) were extracted using an in-house developed script. A set of 3224 molecular descriptors were calculated from optimized molecular structures using Dragon software (version 5.5) (Talete 2007). The molecular descriptors obtained from Dragon are comprised of 22 classes as follows: Constitutional descriptors, Topological descriptors, Walk and path counts, Connectivity indices, Information indices, 2D autocorrelation, Edge adjacency indices, Burden eigenvalues, Topological charge indices, Eigenvalue-based indices, Randic molecular profiles, Geometrical descriptors, RDF descriptors, 3D-MoRSE descriptors, WHIM descriptors, GETAWAY descriptors, Functional group counts, Atom-centred fragments, Charge descriptors, Molecular properties, 2D binary fingerprints and 2D frequency fingerprints.

### Feature selection

The correlation-based feature selection was performed to select informative descriptors from a large set of calculated descriptors. Initially, the pair-correlation of each descriptor value and bioactivity (pIC_50_) was calculated from an initial set of descriptors consisting of 13 quantum chemical descriptors and 3224 molecular descriptors. The correlation coefficient (r) value of 0.6 was used as a cut-off value for initial selection. Descriptors with |r| < 0.6 were considered as low correlated descriptors and were excluded from the study whereas those with |r| ≥ 0.6 were selected for further selection process. The remaining descriptors along with their bioactivity (pIC_50_) were used as an input data for feature selection by stepwise multiple linear regression (MLR) as implemented in SPSS statistics 18.0 (SPSS statistics 18.0 2009). Finally, a set of important descriptors were obtained for multivariate analysis using MLR.

### Multivariate analysis using MLR

Multivariate analysis was performed by MLR using selected descriptors as independent variables (*X*) and pIC_50_ values as dependent variable (*Y*). The MLR models were constructed by Waikato Environment for Knowledge Analysis (WEKA) version 3.4.5 (Witten et al. 2011) according to the following equation:

5 $$\it Y = B_{0} + \sum {BnXn}$$

where *Y* is the pIC_50_ values of compounds, *B*_*0*_ is the intercept and *B*_*n*_ are the regression coefficient of descriptors *X*_*n*_.

### Data sampling

The data set was divided into 2 subsets i.e., training set and testing set by means of leave-one-out cross validation (LOO-CV). Conceptually, one sample was removed from the whole data set (*N*) and were used as the testing set while the remaining samples (*N*-1) were used as the training set. The same process was continued until every sample in the data set was iteratively used as the testing set to predict *Y* variable.

### Evaluating the performance of QSAR models

The predictive performances of the QSAR models were assessed by two statistical parameters i.e., correlation coefficient (*R*) and root mean square error (RMSE). The first parameter (*R*) represented the predictive performance whereas the later (RMSE) represented predictive error of the models.

### Prediction of structurally modified compounds by the constructed QSAR models

Regarding the obtained QSAR equations, the descriptor values of 64 structurally modified compounds (**1A**–**1R**, **2A**–**2R**, **7A**–**7R** and **8A**–**8R**) obtained from Gaussian and Dragon calculations were used as independent variable (*X*) for computing their predicted anticancer activity (pIC_50_ values) against the four tested cancer cell lines.

## Additional files

10.1186/s40064-015-1352-5 Analytical data of the reported compounds.
10.1186/s40064-015-1352-5 Values of informative molecular descriptors of tested (**1**-**32**) and virtually modified compounds (**1A**-**1R**, **2A**-**2R**, **7A-7R** and **8A-8R**).
10.1186/s40064-015-1352-5 Predicted cytotoxic activity (pIC_50_) of modified compounds (**1A**-**1R**, **2A**-**2R**, **7A-7R** and **8A-8R)** and experimental cytotoxic activity of reference drugs.
10.1186/s40064-015-1352-5 A summary of substituent effects on cytotoxic activity of four cancer cell lines.
10.1186/s40064-015-1352-5 Comparison of *meta*- and *para*-triazole derivatives focused on the modified compound series with the best improved activity.
