# Supplementary material for: Discovery of novel 1,2,3-triazole derivatives as anticancer agents using QSAR and in silico structural modification
Source: Springerplus. 2015 Oct 5;4:571. doi: 10.1186/s40064-015-1352-5 (PMC4628044; doi:10.1186/s40064-015-1352-5)
Supplement: Supplementary file 1 — 10.1186/s40064-015-1352-5 Analytical data of the reported compounds. [file 40064_2015_1352_MOESM1_ESM.pdf]

Original Article: *SpringerPlus*

Discovery of novel 1,2,3-triazole derivatives as anticancer agents using QSAR and *in silico* structural modification

Veda Prachayasittikul[1,2], Ratchanok Pingaew[3], Nuttapat Anuwongcharoen[1,2] , Apilak Worachartcheewan[2,4], Chanin Nantasenamat[2], Supaluk Prachayasittikul[2]*, Somsak Ruchirawat[5,6,7]
Virapong Prachayasittikul[1]*

[1]*Department of Clinical Microbiology and Applied Technology, Faculty of Medical Technology, Mahidol University, Bangkok 10700, Thailand*

[2]*Center of Data Mining and Biomedical Informatics, Faculty of Medical Technology, Mahidol University, Bangkok 10700, Thailand*

[3]*Department of Chemistry, Faculty of Science, Srinakharinwirot University, Bangkok 10110, Thailand*

[4]*Department of Clinical Chemistry, Faculty of Medical Technology, Mahidol University, Bangkok 10700, Thailand*

[5]*Laboratory of Medicinal Chemistry, Chulabhorn Research Institute, Bangkok 10210, Thailand*

[6]*Program in Chemical Biology, Chulabhorn Graduate Institute, Bangkok 10210, Thailand*

[7]*Center of Excellence on Environmental Health and Toxicology, Commission on Higher Education (CHE), Ministry of Education, Thailand*

---

*Corresponding authors:

E-mail: virapong.pra@mahidol.ac.th; Telephone: 66-2-441-4376, Fax: 66-2-441-4380

E-mail: supaluk@swu.ac.th; Telephone: 66-2-441-4376, Fax: 66-2-441-4380

Analytical data of the reported compounds

Chemistry

^{1}H- and ^{13}C- NMR spectra were recorded on a Bruker AVANCE 300 NMR spectrometer (operating at 300 MHz for 1H and 75 MHz for ^{13}C). FTIR spectra were obtained using a universal attenuated total reflectance attached on a Perkin–Elmer Spectrum One spectrometer. Mass spectra were recorded on a Bruker Daltonics (microTOF). Melting points were determined using a Griffin melting point apparatus and were uncorrected.

*N-phenethyl-4-(4-phenyl-1H-1,2,3-triazol-1-yl)benzenesulfonamide (**1**)*

White solid. mp 219-220 °C. IR (UATR) cm^{-1}: 3315, 1597, 1504, 1328, 1153. ^{1}H NMR (300 MHz, DMSO-d_6) δ 2.71 (t, *J* = 7.5 Hz, 2H, ArCH_2), 3.04 (q, *J* = 6.9 Hz, 2H, CH_2NH), 7.13-7.30 (m, 5H, Ar*H*), 7.41 (t, *J* = 7.5 Hz, 1H, Ar*H*), 7.52 (t, *J* = 7.2 Hz, 2H, Ar*H*), 7.87-8.05 (m, 5H, Ar*H* and N*H*SO_2), 8.17 (d, *J* = 8.7 Hz, 2H, Ar*H*), 9.42 (s, 1H, C*H*N). ^{13}C NMR (75 MHz, DMSO-d_6) δ 35.7, 44.5, 120.3, 120.9, 125.9, 126.7, 128.8, 128.9, 129.1, 129.5, 130.4, 139.1, 139.5, 140.6, 148.1. HRMS-TOF: m/z $[M + H]^+$ 405.1371 (Calcd for $C_{22}H_{21}N_4O_2S$: 405.1380).

*N-phenethyl-4-(4-(phenoxymethyl)-1H-1,2,3-triazol-1-yl)benzenesulfonamide (**2**)*

White solid. mp 149-150 °C. IR (UATR) cm^{-1}: 3278, 1597, 1496, 1331, 1159. ^{1}H NMR (300 MHz, $CDCl_3$) δ 2.83 (t, *J* = 6.8 Hz, 2H, ArCH_2), 3.31 (q, *J* = 6.8 Hz, 2H, CH_2NH), 4.56 (t, *J* = 6.1 Hz, 1H, N*H*SO_2), 5.34 (s, 2H, CH_2O), 7.00-7.38 (m, 10H, Ar*H*), 7.90 (d, *J* = 8.9 Hz, 2H, Ar*H*), 7.97 (d, *J* = 8.9 Hz, 2H, Ar*H*), 8.15 (s, 1H, C*H*N). ^{13}C NMR (75 MHz, $CDCl_3$) δ 35.8, 44.3, 61.9, 114.8, 120.6, 121.6, 127.0, 128.7, 128.9, 129.7, 137.3, 139.7, 140.2, 145.9, 158.0. HRMS-TOF: m/z $[M + H]^+$ 435.1473 (Calcd for $C_{23}H_{23}N_4O_3S$: 435.1496).

*4-(4-((naphthalen-2-yloxy)methyl)-1H-1,2,3-triazol-1-yl)-N-phenethylbenzenesulfonamide (**3**)*

White solid. mp 179-180 °C. IR (UATR) cm^{-1}: 3247, 1598 , 1505, 1312, 1154. ^{1}H NMR (300 MHz, DMSO-d_6) δ 2.70 (t, *J* = 7.6 Hz, 2H, ArCH_2), 3.03 (q, *J* = 7.0 Hz, 2H, CH_2NH), 5.39 (s, 2H, CH_2O), 7.12-7.29 (m, 6H, Ar*H*), 7.37 (t, *J* = 8.1 Hz, 1H, Ar*H*), 7.49 (t, *J* = 7.9 Hz, 1H, Ar*H*), 7.56 (d, *J* = 2.2 Hz, 1H, Ar*H*), 7.81-7.95 (m, 4H, Ar*H* and N*H*SO_2), 7.98 (d, *J* = 8.6 Hz, 2H, Ar*H*), 8.16 (d, *J* = 8.7 Hz, 2H, Ar*H*), 9.14 (s, 1H, C*H*N). ^{13}C NMR (75 MHz, DMSO-d_6) δ 35.7, 44.5, 61.5, 107.8, 119.1, 121.1, 123.7, 124.3, 126.7, 127.0, 127.3, 128.0, 128.8, 128.9, 129.1, 129.9, 134.6, 139.1, 139.4, 140.7, 144.7, 156.3. HRMS-TOF: m/z $[M + H]^+$ 485.1635 (Calcd for $C_{27}H_{25}N_4O_3S$: 485.1642).

*N-phenethyl-4-(4-((p-tolyloxy)methyl)-1H-1,2,3-triazol-1-yl)benzenesulfonamide (**4**)*

White solid. mp 150-151 ºC. IR (UATR) cm^{-1}: 3273, 1597, 1508, 1331, 1159. ^1H NMR (300 MHz, $CDCl_3$) δ 2.32 (s, 3H, ArCH_3), 2.82 (t, *J* = 6.8 Hz, 2H, ArCH_2), 3.30 (q, *J* = 6.4 Hz, 2H, CH_2NH), 4.55 (br. s, 1H, N*H*SO_2), 5.31 (s, 2H, CH_2O), 6.94 (d, *J* = 8.6 Hz, 2H, Ar*H*), 7.08-7.17 (m, 4H, Ar*H*), 7.22-7.33 (m, 3H, Ar*H*), 7.90 (d, *J* = 8.8 Hz, 2H, Ar*H*), 7.97 (d, *J* = 8.8 Hz, 2H, Ar*H*), 8.14 (s, 1H, C*H*N). ^{13}C NMR (75 MHz, $CDCl_3$) δ 20.5, 35.8, 44.3, 62.0, 114.6, 120.6, 127.0, 128.7, 128.9, 130.1, 130.9, 137.3, 139.7, 140.2, 146.0, 155.9. HRMS-TOF: m/z $[M + H]^+$ 449.1641 (Calcd for $C_{24}H_{25}N_4O_3S$: 449.1642).

*4-(4-((4-formylphenoxy)methyl)-1H-1,2,3-triazol-1-yl)-N-phenethylbenzenesulfonamide (**5**)*

White solid. mp 141-142 ºC. IR (UATR) cm^{-1}: 3267, 1687, 1597, 1507 , 1330, 1158. ^1H NMR (300 MHz, $CDCl_3$) δ 2.83 (t, *J* = 6.8 Hz, 2H, ArCH_2), 3.31 (q, *J* = 6.8 Hz, 2H, CH_2NH), 4.62 (t, *J* = 6.1 Hz, 1H, N*H*SO_2), 5.42 (s, 2H, CH_2O), 7.08-7.34 (m, 7H, Ar*H*), 7.85-8.01 (m, 6H, Ar*H*), 8.19 (s, 1H, C*H*N), 9.92 (s, 1H, C*H*O). ^{13}C NMR (75 MHz, $CDCl_3$) δ 35.8, 44.3, 62.0, 115.1, 120.7, 120.9, 127.0, 128.7, 128.9, 129.0, 130.6, 132.1, 137.3, 139.5, 140.5, 144.8, 162.9, 190.7. HRMS-TOF: m/z $[M + H]^+$ 463.1428 (Calcd for $C_{24}H_{23}N_4O_4S$: 463.1434).

*4.4.2 4-(4-((4-nitrophenoxy)methyl)-1H-1,2,3-triazol-1-yl)-N-phenethylbenzenesulfonamide (**6**)*

White solid. mp 192-193 ºC. IR (UATR) cm^{-1}: 3280, 1590, 1506, 1338, 1158. ^1H NMR (300 MHz, DMSO-d_6) δ 2.70 (t, *J* = 7.6 Hz, 2H, ArCH_2), 3.03 (q, *J* = 6.9 Hz, 2H, CH_2NH), 5.45 (s, 2H, CH_2O), 7.13-7.35 (m, 7H, Ar*H*), 7.91 (t, *J* = 5.7 Hz, 1H, N*H*SO_2), 7.98 (d, *J* = 8.7 Hz, 2H, Ar*H*), 8.14 (d, *J* = 8.7 Hz, 2H, Ar*H*), 8.25 (d, *J* = 9.0 Hz, 2H, Ar*H*), 9.11 (s, 1H, C*H*N). ^{13}C NMR (75 MHz, DMSO-d_6) δ 35.7, 44.5, 62.2, 115.9, 121.2, 124.0, 126.4, 126.7, 128.8, 128.9, 129.1, 139.0, 139.3, 140.8, 141.7, 143.8, 163.6. HRMS-TOF: m/z $[M + H]^+$ 480.1337 (Calcd for $C_{23}H_{22}N_5O_5S$: 480.1336).

*N-phenethyl-4-(4-((o-tolyloxy)methyl)-1H-1,2,3-triazol-1-yl)benzenesulfonamide (**7**)*

White solid. mp 172-173 ºC. IR (UATR) cm^{-1}: 3187, 1594, 1496, 1333, 1161. ^1H NMR (300 MHz, DMSO-d_6) δ 2.16 (s, 3H, ArCH_3), 2.70 (t, J=7.6 Hz, 2H, ArCH_2), 3.02 (q, J=7.1 Hz, 2H, CH_2NH), 5.26 (s, 2H, CH_2O), 6.83-6.90 (m, 1H, Ar*H*), 7.12-7.29 (m, 8H, Ar*H*), 7.91 (t, J=5.7 Hz, 1H, N*H*SO_2), 7.98 (d, J=8.8 Hz, 2H, Ar*H*), 8.15 (d, J=8.8 Hz, 2H, Ar*H*), 9.06 (s, 1H, C*H*N). ^{13}C NMR (75 MHz, DMSO-d_6) δ 16.5, 35.7, 44.5, 61.7, 112.4, 121.1, 121.2, 123.3, 126.5, 126.7, 127.4 , 128.8, 129.1, 131.0, 139.0, 139.4, 140.7, 145.2, 156.5. HRMS-TOF: m/z $[M + H]^+$ 449.1629 (Calcd for $C_{24}H_{25}N_4O_3S$: 449.1642).

*methyl 2-((1-(4-(N-phenethylsulfamoyl)phenyl)-1H-1,2,3-triazol-4-yl)methoxy)benzoate (**8**)*

White solid. mp 135-136 °C. IR (UATR) cm^{-1}: 3275, 1717, 1599, 1490, 1307, 1159. ^{1}H NMR (300 MHz, $CDCl_3$) δ 2.82 (t, *J* = 6.8 Hz, 2H, ArCH_2), 3.31 (br t, 2H, CH_2NH), 3.92 (s, 3H, CO_2CH_3), 4.62 (br s, 1H, N*H*SO_2), 5.44 (s, 2H, CH_2O), 7.04-7.33 (m, 7H, Ar*H*), 7.49-7.57 (m, 1H, Ar*H*), 7.85-8.00 (m, 5H, Ar*H*), 8.36 (s, 1H, C*H*N). ^{13}C NMR (75 MHz, $CDCl_3$) δ 35.8, 44.3, 52.0, 63.4, 114.1, 120.6, 121.1, 121.3, 127.0, 128.7, 128.9, 131.8, 133.8, 137.4, 139.7, 140.1, 157.8, 166.2. HRMS-TOF: m/z [M + Na]$^+$ 515.1359 (Calcd for $C_{25}H_{24}N_4NaO_5S$: 515.1371).

*4-(4-((4-formyl-2-methoxyphenoxy)methyl)-1H-1,2,3-triazol-1-yl)-N-phenethylbenzenesulfonamide (**9**)*

Pale yellow solid. mp 100-101 °C. IR (UATR) cm^{-1}: 3300, 1677, 1586, 1504, 1334, 1156. ^{1}H NMR (300 MHz, $CDCl_3$) δ 2.78 (t, *J* = 6.8 Hz, 2H, ArCH_2), 3.26 (q, *J* = 6.7 Hz, 2H, CH_2NH), 3.92 (s, 3H, OCH_3), 4.67 (t, *J* = 6.1 Hz, 1H, N*H*SO_2), 5.44 (s, 2H, CH_2O), 7.06 (dd, *J* = 7.9, 1.7 Hz, 2H, Ar*H*), 7.18-7.28 (m, 4H, Ar*H*), 7.40-7.47 (m, 2H, Ar*H*), 7.85 (d, *J* = 8.8 Hz, 2H, Ar*H*), 7.92 (d, *J* = 8.7 Hz, 2H, Ar*H*), 8.18 (s, 1H, C*H*N), 9.84 (s, 1H, C*H*O). ^{13}C NMR (75 MHz, $CDCl_3$) δ 35.8, 44.3, 56.1, 62.7, 109.5, 112.6, 120.7, 121.2, 126.6, 127.0, 128.7, 128.9, 130.9, 137.4, 139.5, 140.4, 150.0, 152.8, 190.9. HRMS-TOF: m/z [M + H]$^+$ 493.1545 (Calcd for $C_{25}H_{25}N_4O_5S$: 493.1540).

*4-(4-((5-formyl-2-methoxyphenoxy)methyl)-1H-1,2,3-triazol-1-yl)-N-phenethylbenzenesulfonamide (**10**)*

Light brown solid. mp 204-205 °C. IR (UATR) cm^{-1}: 3304, 1686, 1583, 1514, 1335, 1162. ^{1}H NMR (300 MHz, DMSO-d_6) δ 2.70 (t, *J* = 6.8 Hz, 2H, ArCH_2), 3.02 (q, *J* = 6.8 Hz, 2H, CH_2NH), 3.87 (s, 3H, OCH_3), 5.33 (s, 2H, CH_2O), 7.13-7.30 (m, 6H, Ar*H*), 7.62 (dd, *J* = 8.2, 1.7 Hz, 1H, Ar*H*), 7.66 (d, *J* = 1.7 Hz, 1H, Ar*H*), 7.90 (t, *J* = 5.7 Hz, 1H, N*H*SO_2), 7.98 (d, *J* = 8.7 Hz, 2H, Ar*H*), 8.15 (d, *J* = 8.7 Hz, 2H, Ar*H*), 9.07 (s, 1H, C*H*N), 9.86 (s, 1H, C*H*O). ^{13}C NMR (75 MHz, DMSO-d_6) δ 35.7, 44.5, 56.4, 62.1, 112.2, 112.3, 121.1, 123.9, 126.7, 126.9, 128.8, 129.1, 130.1, 139.1, 139.4, 140.8, 144.3, 148.2, 155.0, 191.8. HRMS-TOF: m/z [M + H]$^+$ 493.1536 (Calcd for $C_{25}H_{25}N_4O_5S$: 493.1540).

*4-(4-(((2-oxo-2H-chromen-7-yl)oxy)methyl)-1H-1,2,3-triazol-1-yl)-N-phenethylbenzenesulfonamide (**11**)*

White solid. mp 130-131 °C. IR (UATR) cm^{-1}: 3246, 1718, 1618, 1595, 1505, 1277, 1153. ^{1}H NMR (300 MHz, $CDCl_3$) δ 2.79 (t, *J* = 6.8 Hz, 2H, ArCH_2), 3.27 (q, *J* = 6.6 Hz, 2H, CH_2NH), 4.60 (t, *J* = 5.8 Hz, 1H, N*H*SO_2), 5.35 (s, 2H, CH_2O), 6.26 (d, *J* = 9.5, 1H, COC*H*=CH), 6.91-6.98 (m, 2H, Ar*H*), 7.07 (d, *J* = 6.6 Hz, 2H, Ar*H*), 7.17-7.29 (m, 3H, Ar*H*), 7.40 (d, *J* = 9.2 Hz, 1H, Ar*H*), 7.63 (d, *J* = 9.5 Hz, 1H, COCH=C*H*), 7.87 (d, *J* = 8.7 Hz, 2H, Ar*H*), 7.93 (d, *J* = 8.7 Hz, 2H, Ar*H*), 8.50 (s, 1H, C*H*N). ^{13}C NMR (75 MHz, $CDCl_3$) δ 35.8, 44.3, 62.2, 102.2, 112.7, 113.2, 113.7, 120.7, 121.0, 127.0, 128.7, 128.9, 129.0,

137.4, 139.5, 140.4, 143.2, 155.8, 161.0, 161.1. HRMS-TOF: m/z $[M + H]^+$ 503.1392 (Calcd for $C_{26}H_{23}N_4O_5S$: 503.1384).

*4-(4-(((2-oxo-2H-chromen-4-yl)oxy)methyl)-1H-1,2,3-triazol-1-yl)-N-phenethylbenzenesulfonamide (**12**)*

White solid. mp 181-182 °C. IR (UATR) cm^{-1}: 3147, 1719, 1624, 1328, 1157. ^1H NMR (300 MHz, DMSO-d_6) δ 2.70 (t, *J* = 6.8 Hz, 2H, ArCH_2), 3.02 (q, *J* = 6.8 Hz, 2H, CH_2NH), 5.56 (s, 2H, CH_2O), 6.22 (s, 1H, COC*H*), 7.13-7.46 (m, 5H, Ar*H*), 7.36 (t, *J* = 8.0, 1H, Ar*H*), 7.43 (d, *J* = 7.8 Hz, 1H, Ar*H*), 7.66 (dt, *J* = 8.0, 1.4 Hz, 1H, Ar*H*), 7.85 (dd, *J* = 7.9, 1.4 Hz, 1H, Ar*H*), 7.92 (t, *J* = 5.7 Hz, 1H, N*H*SO_2), 7.99 (d, *J* = 8.8 Hz, 2H, Ar*H*), 8.18 (d, *J* = 8.8 Hz, 2H, Ar*H*), 9.21 (s, 1H, C*H*N). ^{13}C NMR (75 MHz, DMSO-d_6) δ 35.7, 44.5, 63.2, 92.0, 115.5, 116.9, 121.2, 123.5, 124.1, 124.7, 126.7, 128.8, 128.9, 133.3, 139.0, 139.3, 140.9, 143.1, 153.3, 162.0, 164.8. HRMS-TOF: m/z $[M + H]^+$ 503.1383 (Calcd for $C_{26}H_{23}N_4O_5S$: 503.1384).

*N-(3,4-dimethoxyphenethyl)-4-(4-phenyl-1H-1,2,3-triazol-1-yl)benzenesulfonamide (**13**)*

Pale yellow solid. mp 147-148 °C. IR (UATR) cm^{-1}: 3271 (NH), 1596 (ar C=C), 1516 (ar C=C), 1330 (S=O), 1157 (S=O). ^1H NMR (300 MHz, $CDCl_3$) δ 2.74 (t, *J* = 6.8 Hz, 2H, ArCH_2), 3.26 (q, *J* = 6.8 Hz, 2H, CH_2NH), 3.78, 3.81 (2s, 6H, 2 × OCH_3), 4.59 (t, *J* = 6.2 Hz, 1H, N*H*SO_2), 6.56 (d, *J* = 1.7 Hz, 1H, C2-Ar*H*), 6.61 (dd, *J* = 8.1,1.7 Hz, 1H, C6-Ar*H*), 6.74 (d, *J* = 8.1 Hz, 1H, C5-Ar*H*), 7.35-7.50 (m, 3H, Ar*H*), 7.87-7.98 (m, 6H, Ar*H*), 8.27 (s, 1H, C*H*N). ^{13}C NMR (75 MHz, $CDCl_3$) δ 35.4, 44.4, 55.9, 56.0, 111.6, 111.9, 117.3, 120.5, 120.8, 126.0, 128.8, 128.9, 129.7, 129.9, 139.7, 140.0, 148.1, 149.0, 149.2. HRMS-TOF: m/z $[M + H]^+$ 465.1590 (Calcd for $C_{24}H_{25}N_4O_4S$: 465.1591).

*N-(3,4-dimethoxyphenethyl)-4-(4-((4-formylphenoxy)methyl)-1H-1,2,3-triazol-1-yl)benzenesulfonamide (**14**)*

Light brown solid. mp 143-144 °C. IR (UATR) cm^{-1}: 3262, 1688, 1597, 1508, 1330, 1159. ^1H NMR (300 MHz, $CDCl_3$) δ 2.73 (t, *J* = 6.5 Hz, 2H, ArCH_2), 3.24 (q, *J* = 6.2 Hz, 2H, CH_2NH), 3.76, 3.80 (2s, 6H, 2 × OCH_3), 4.58 (t, *J* = 6.0 Hz, 1H, N*H*SO_2), 5.38 (s, 2H, CH_2O), 6.55 (br d, 1H, C2-Ar*H*), 6.60 (d, *J* = 7.8 Hz, 1H, C6-Ar*H*), 6.73 (d, *J* = 8.1 Hz, 1H, C5-Ar*H*), 7.81-7.96 (m, 6H, Ar*H*), 7.12 (d, *J* = 8.6 Hz, 2H, Ar*H*), 8.17 (s, 1H, C*H*N), 9.88 (s, 1H, C*H*O). ^{13}C NMR (75 MHz, $CDCl_3$) δ 35.4, 44.4, 55.8, 55.9, 62.0, 111.5, 111.8, 115.1, 120.6, 120.7, 120.8, 121.0, 128.9, 129.8, 130.6, 132.0, 139.5, 140.4, 144.7, 148.1, 149.2, 162.9, 190.7. HRMS-TOF: m/z $[M + H]^+$ 523.1650 (Calcd for $C_{26}H_{27}N_4O_6S$: 523.1646).

*N-(3,4-dimethoxyphenethyl)-4-(4-((4-nitrophenoxy)methyl)-1H-1,2,3-triazol-1-yl)benzenesulfonamide (**15**)*

Light brown solid. mp 183-184 ºC. IR (UATR) cm^{-1}: 3257, 1593, 1519, 1343, 1160. ^{1}H NMR (300 MHz, DMSO-d_6) δ 2.62 (t, *J* = 7.4 Hz, 2H, ArC*H*$_2$), 3.03 (q, *J* = 7.0 Hz, 2H, C*H*$_2$NH), 3.65, 3.68 (2s, 6H, 2 × OC*H*$_3$), 5.45 (s, 2H, C*H*$_2$O), 6.64 (dd, *J* = 8.1, 1.8 Hz, 1H, C6-Ar*H*), 6.72 (d, *J* = 1.7 Hz, 1H, C2-Ar*H*), 6.78 (d, *J* = 8.2 Hz, 1H, C5-Ar*H*), 7.32 (d, *J* = 9.3 Hz, 2H, C2″-Ar*H* and C6″-Ar*H*), 7.85 (t, *J* = 5.6 Hz, 1H, N*H*SO$_2$), 7.94 (d, *J* = 8.7 Hz, 2H, C3′-Ar*H* and C5′-Ar*H*), 8.11 (d, *J* = 8.7 Hz, 2H, C2′-Ar*H* and C6′-Ar*H*), 8.25 (d, *J* = 9.2 Hz, 2H, C3″-Ar*H* and C5″-Ar*H*), 9.09 (s, 1H, C*H*N). ^{13}C NMR (75 MHz, DMSO-d_6) δ 35.3, 44.7, 55.8, 55.9, 62.2, 112.2, 113.0, 115.9, 121.0, 123.9, 126.4, 128.8, 131.4, 139.2, 140.9, 141.7, 143.8, 147.8, 149.0, 163.6. HRMS-TOF: m/z $[M + H]^+$ 540.1563 (Calcd for $C_{25}H_{26}N_5O_7S$: 540.1548).

*(1-(4-((3,4-dihydroisoquinolin-2(1H)-yl)sulfonyl)phenyl)-1H-1,2,3-triazol-4-yl)methanol (**16**)*

White solid. mp 111-112 ºC. IR (UATR) cm^{-1}: 3280, 1597, 1503, 1336, 1243, 1161. ^{1}H NMR (300 MHz, DMSO-d_6) *δ* 2.87 (br t, 2H, C4-*H*), 3.35 (br t, 2H, C3-*H*), 4.27 (s, 2H, C1-*H*), 4.62 (d, *J* = 5.4 Hz, 2H, C*H*$_2$OH), 5.41 (t, *J* = 5.4 Hz, 1H, CH$_2$O*H*), 7.05-7.20 (m, 4H, Ar*H*), 8.03 (d, *J* = 8.7 Hz, 2H, Ar*H*), 7.97 (d, *J* = 8.6 Hz, 2H, Ar*H*), 8.85 (s, 1H, C*H*N). ^{13}C NMR (75 MHz, DMSO-d_6) *δ* 28.4, 44.0, 47.7, 55.4, 120.8, 121.7, 126.6, 126.9, 127.2, 129.1, 129.8, 132.0, 133.4, 136.0, 140.2, 150.1. HRMS-TOF: m/z $[M + Na]^+$ 393.0988 (Calcd for $C_{18}H_{18}N_4NaO_3S$: 393.0992).

*2-((4-(4-(phenoxymethyl)-1H-1,2,3-triazol-1-yl)phenyl)sulfonyl)-1,2,3,4-tetrahydroisoquinoline (**17**)*

White solid. mp 186-187 ºC. IR (UATR) cm^{-1}: 1598, 1497, 1337, 1243, 1162. ^{1}H NMR (300 MHz, $CDCl_3$) *δ* 2.92 (t, *J* = 6.0 Hz, 2H, C4-*H*), 3.43 (t, *J* = 6.0 Hz, 2H, C3-*H*), 4.32 (s, 2H, C1-*H*), 5.29 (s, 2H, C*H*$_2$O), 6.94-7.33 (m, 9H, Ar*H*), 7.91 (d, *J* = 8.8 Hz, 2H, Ar*H*), 7.98 (d, *J* = 8.8 Hz, 2H, Ar*H*), 8.10 (s, 1H, C*H*N). ^{13}C NMR (75 MHz, $CDCl_3$) *δ* 28.7, 43.7, 47.5, 61.9, 114.7, 120.6, 121.5, 126.3, 126.5, 127.0, 128.9, 129.4, 129.7, 131.2, 132.9, 137.1, 139.9, 145.9, 158.0. HRMS-TOF: m/z $[M + Na]^+$ 469.1292 (Calcd for $C_{24}H_{22}N_4NaO_3S$: 469.1305).

*2-((4-(4-((naphthalen-2-yloxy)methyl)-1H-1,2,3-triazol-1-yl)phenyl)sulfonyl)-1,2,3,4-tetrahydroisoquinoline (**18**)*

White solid. mp 241-242 ºC. IR (UATR) cm^{-1}: 1597, 1508, 1337, 1243, 1161. ^{1}H NMR (300 MHz, $CDCl_3$) *δ* 2.91 (t, *J* = 5.6 Hz, 2H, C4-*H*), 3.43 (t, *J* = 5.8 Hz, 2H, C3-*H*), 4.32 (s, 2H, C1-*H*), 5.26 (s, 2H, C*H*$_2$O), 6.85-7.18 (m, 11H, Ar*H*), 7.91 (d, *J* = 8.6 Hz, 2H, Ar*H*), 7.98 (d, *J* = 8.6 Hz, 2H, Ar*H*), 8.09 (s, 1H, C*H*N). ^{13}C NMR (75 MHz, $CDCl_3$) *δ* 28.7, 43.7, 47.5, 62.1, 114.6, 120.6, 126.3, 126.5, 127.0, 128.9,

129.4, 130.1, 130.9, 131.2, 132.9, 137.2, 139.9, 146.1, 156.0. HRMS-TOF: m/z $[M + H]^+$ 497.1647 (Calcd for $C_{28}H_{25}N_4O_3S$: 497.1653).

2-((4-(4-((o-tolyloxy)methyl)-1H-1,2,3-triazol-1-yl)phenyl)sulfonyl)-1,2,3,4-tetrahydroisoquinoline ***(19)***

White solid. mp 168-169 °C. IR (UATR) cm^{-1}: 1596, 1495, 1339, 1240, 1164. ^{1}H NMR (300 MHz, $CDCl_3$) δ 2.28 (s, 3H, C*H*$_3$), 2.96 (t, *J* = 5.9 Hz, 2H, C4-*H*), 3.47 (t, *J* = 5.9 Hz, 2H, C3-*H*), 4.36 (s, 2H, C1-*H*), 5.34 (s, 2H, C*H*$_2$O), 6.90-7.24 (m, 8H, Ar*H*), 7.95 (d, *J* = 8.9 Hz, 2H, Ar*H*), 8.03 (d, *J* = 8.8 Hz, 2H, Ar*H*), 8.11 (s, 1H, C*H*N). ^{13}C NMR (75 MHz, $CDCl_3$) δ 16.3, 28.7, 43.7, 47.5, 62.2, 111.5, 120.4, 120.6, 121.3, 126.3, 126.5, 127.0, 128.9, 129.4, 131.0, 131.2, 132.9, 139.9, 146.3, 156.2. HRMS-TOF: m/z $[M + H]^+$ 461.1637 (Calcd for $C_{25}H_{25}N_4O_3S$: 461.1653).

1-(2-((1-(4-((3,4-dihydroisoquinolin-2(1H)-yl)sulfonyl)phenyl)-1H-1,2,3-triazol-4-yl)methoxy)phenyl)ethanone ***(20)***

White solid. mp 185-186 °C. IR (UATR) cm^{-1}: 1655, 1595, 1450, 1358, 1161. ^{1}H NMR (300 MHz, $CDCl_3$) δ 2.58 (s, 3H, COC*H*$_3$), 2.91 (t, *J* = 6.0 Hz, 2H, C4-*H*), 3.42 (t, *J* = 6.0 Hz, 2H, C3-*H*), 4.31 (s, 2H, C1-*H*), 5.38 (s, 2H, C*H*$_2$O), 6.99-7.15 (m, 6H, Ar*H*), 7.46 (dt, *J* = 7.7, 1.7 Hz, 1H, Ar*H*), 7.69 (dd, *J* = 7.7, 1.7 Hz, 1H, Ar*H*), 7.92 (d, *J* = 8.8 Hz, 2H, Ar*H*), 7.99 (d, *J* = 8.8 Hz, 2H, Ar*H*), 8.15 (s, 1H, C*H*N). ^{13}C NMR (75 MHz, $CDCl_3$) δ 28.7, 31.7, 43.7, 47.5, 62.4, 113.1, 120.7, 120.8, 121.5, 126.3, 126.5, 127.0, 128.9, 129.0, 129.5, 130.5, 131.2, 132.9, 133.6, 137.2, 139.7, 145.1, 157.0, 199.7. HRMS-TOF: m/z $[M + Na]^+$ 511.1391 (Calcd for $C_{26}H_{24}N_4NaO_4S$: 511.1410).

methyl 2-((1-(4-((3,4-dihydroisoquinolin-2(1H)-yl)sulfonyl)phenyl)-1H-1,2,3-triazol-4-yl)methoxy)benzoate ***(21)***

White solid. mp 162-163 °C. IR (UATR) cm^{-1}: 1696, 1599, 1453, 1306, 1254, 1160. ^{1}H NMR (300 MHz, $CDCl_3$) δ 2.92 (t, *J* = 6.0 Hz, 2H, C4-*H*), 3.43 (t, *J* = 6.0 Hz, 2H, C3-*H*), 3.88 (s, 3H, CO$_2$C*H*$_3$), 4.32 (s, 2H, C1-*H*), 5.39 (s, 2H, C*H*$_2$O), 7.00-7.18 (m, 6H, Ar*H*), 7.48 (dt, *J* = 7.8, 1.7 Hz, 1H, Ar*H*), 7.82 (dd, *J* = 7.8, 1.7 Hz, 1H, Ar*H*), 7.93 (d, *J* = 8.9 Hz, 2H, Ar*H*), 7.99 (d, *J* = 8.9 Hz, 2H, Ar*H*), 8.31 (s, 1H, C*H*N). ^{13}C NMR (75 MHz, $CDCl_3$) δ 28.7, 43.7, 47.5, 52.0, 63.4, 114.1, 120.6, 121.0, 121.3, 126.3, 126.5, 127.0, 128.9, 129.4, 131.2, 131.8, 132.9, 133.8, 137.0, 139.9, 146.0, 157.8, 166.1. HRMS-TOF: m/z $[M + Na]^+$ 527.1365 (Calcd for $C_{26}H_{24}N_4NaO_5S$: 527.1360).

4-((1-(4-((3,4-dihydroisoquinolin-2(1H)-yl)sulfonyl)phenyl)-1H-1,2,3-triazol-4-yl)methoxy)benzaldehyde ***(22)***

White solid. mp 189-190 ºC. IR (UATR) cm^{-1}: 1686, 1605, 1578, 1500, 1357, 1243, 1160. ^{1}H NMR (300 MHz, $CDCl_3$) δ 2.92 (t, J = 5.9 Hz, 2H, C4-*H*), 3.43 (t, J = 5.9 Hz, 2H, C3-*H*), 4.32 (s, 2H, C1-*H*), 5.37 (s, 2H, CH_2O), 7.00-7.18 (m, 6H, Ar*H*), 7.84 (d, J = 8.8 Hz, 2H, Ar*H*), 7.92 (d, J = 8.8 Hz, 2H, Ar*H*), 7.99 (d, J = 8.8 Hz, 2H, Ar*H*), 8.14 (s, 1H, C*H*N), 9.88 (s, 3H, C*H*O). ^{13}C NMR (75 MHz, $CDCl_3$) δ 28.7, 43.7, 47.5, 62.0, 115.1, 120.7, 120.9, 126.3, 126.5, 127.0, 128.9, 129.5, 130.6, 131.2, 132.2, 132.8, 137.4, 139.7, 144.8, 162.9, 190.7. HRMS-TOF: m/z $[M + H]^+$ 475.1450 (Calcd for $C_{25}H_{23}N_4O_4S$: 475.1446).

4-((1-(4-((3,4-dihydroisoquinolin-2(1H)-yl)sulfonyl)phenyl)-1H-1,2,3-triazol-4-yl)methoxy)-2H-chromen-2-one ***(23)***

White solid. mp 150-151 ºC. IR (UATR) cm^{-1}: 1687, 1618, 1456, 1354, 1248, 1161. ^{1}H NMR (300 MHz, $CDCl_3$) δ 2.96 (t, J = 5.9 Hz, 2H, C4-*H*), 3.47 (t, J = 5.9 Hz, 2H, C3-*H*), 4.36 (s, 2H, C1-*H*), 5.46 (s, 2H, CH_2O), 5.92 (s, 1H, C*H*CO), 7.04-7.36 (m, 6H, Ar*H*), 7.57 (dt, J = 7.7, 1.4 Hz, 1H, Ar*H*), 7.82 (dd, J = 7.9, 1.4 Hz, 1H, Ar*H*), 7.99 (d, J = 8.7 Hz, 2H, Ar*H*), 8.05 (d, J = 8.7 Hz, 2H, Ar*H*), 8.27 (s, 1H, C*H*N). ^{13}C NMR (75 MHz, $CDCl_3$) δ 28.7, 43.7, 47.5, 62.4, 91.4, 115.4, 116.8, 120.8, 121.5, 123.1, 124.0, 126.3, 126.6, 127.0, 128.9, 129.5, 131.2, 132.7, 132.8, 139.6, 142.9, 153.4, 162.4, 164.8. HRMS-TOF: m/z $[M + H]^+$ 515.1384 (Calcd for $C_{27}H_{23}N_4O_5S$: 515.1384).

(1-(4-((6,7-dimethoxy-3,4-dihydroisoquinolin-2(1H)-yl)sulfonyl)phenyl)-1H-1,2,3-triazol-4-yl)methanol ***(24)***

Pale yellow solid. mp 159-160 ºC. IR (UATR) cm^{-1}: 3280, 1599, 1523, 1342, 1226, 1158. ^{1}H NMR (300 MHz, DMSO-d_6) δ 2.78 (t, J = 5.5 Hz, 2H, C4-*H*), 3.33 (t, J = 5.8 Hz, 2H, C3-*H*), 3.67, 3.68 (2s, 6H, 2 × OCH_3), 4.16 (s, 2H, C1-*H*), 4.62 (d, J = 5.5 Hz, 2H, CH_2OH), 5.41 (t, J = 5.4 Hz, 1H, CH_2O*H*), 6.67 (s, 1H, Ar*H*), 6.76 (s, 1H, Ar*H*), 7.99 (d, J = 8.8 Hz, 2H, Ar*H*), 8.19 (d, J = 8.8 Hz, 2H, Ar*H*), 8.83 (s, 1H, C*H*N). ^{13}C NMR (75 MHz, DMSO-d_6) δ 28.0, 44.1, 47.4, 55.4, 55.9, 56.0, 110.3, 112.3, 120.8, 121.7, 123.6, 125.2, 129.8, 136.0, 140.2, 147.8, 148.1, 150.1. HRMS-TOF: m/z $[M + H]^+$ 431.1389 (Calcd for $C_{20}H_{23}N_4O_5S$: 431.1384).

6,7-dimethoxy-2-((4-(4-(phenoxymethyl)-1H-1,2,3-triazol-1-yl)phenyl)sulfonyl)-1,2,3,4-tetrahydroisoquinoline ***(25)***

White solid. mp 158-159 ºC. IR (UATR) cm^{-1}: 1597, 1519, 1348, 1227, 1163. ^{1}H NMR (300 MHz, $CDCl_3$) δ 2.82 (t, J = 6.0 Hz, 2H, C4-*H*), 3.40 (t, J = 6.0 Hz, 2H, C3-*H*), 3.80 (s, 6H, 2 × OCH_3), 4.24 (s, 2H, C1-*H*), 5.29 (s, 2H, CH_2O), 6.49 (s, 1H, Ar*H*), 6.53 (s, 1H, Ar*H*), 6.94-7.02 (m, 3H, Ar*H*), 7.24-7.34

(m, 2H, Ar*H*), 7.91 (d, *J* = 8.8 Hz, 2H, Ar*H*), 7.98 (d, *J* = 8.8 Hz, 2H, Ar*H*), 8.10 (s, 1H, C*H*N). ^{13}C NMR (75 MHz, $CDCl_3$) δ 28.2, 43.8, 47.2, 55.9, 56.0, 61.9, 109.0, 111.4, 114.7, 120.6, 121.6, 123.0, 124.8, 129.4, 129.7, 137.2, 139.8, 145.9, 147.9, 148.1, 158.0. HRMS-TOF: m/z $[M + H]^+$ 507.1706 (Calcd for $C_{26}H_{27}N_4O_5S$: 507.1697).

*6,7-dimethoxy-2-((4-(4-((naphthalen-2-yloxy)methyl)-1H-1,2,3-triazol-1-yl)phenyl)sulfonyl)-1,2,3,4-tetrahydroisoquinoline (**26**)*

Pale yellow solid. mp 210-211 °C. IR (UATR) cm^{-1}: 1598, 1519, 1463, 1347, 1257, 1163. ^{1}H NMR (300 MHz, $CDCl_3$) δ 2.86 (t, *J* = 5.7 Hz, 2H, C4-*H*), 3.43 (t, *J* = 5.7 Hz, 2H, C3-*H*), 3.83, 3.84 (2s, 6H, 2 × OCH_3), 4.27 (s, 2H, C1-*H*), 5.45 (s, 2H, CH_2O), 6.53 (s, 1H, Ar*H*), 6.56 (s, 1H, Ar*H*), 7.22 (dd, *J* = 8.9, 2.5 Hz, 1H, Ar*H*), 7.31 (d, *J* = 2.4 Hz, 1H, Ar*H*), 7.38 (t, *J* = 8.1 Hz, 1H, Ar*H*), 7.47 (t, *J* = 8.1 Hz, 1H, Ar*H*), 7.75-7.82 (m, 3H, Ar*H*), 7.95 (d, *J* = 8.9 Hz, 2H, Ar*H*), 8.01 (d, *J* = 8.9 Hz, 2H, Ar*H*), 8.17 (s, 1H, C*H*N). ^{13}C NMR (75 MHz, $CDCl_3$) δ 28.2, 43.8, 47.2, 55.9, 56.0, 61.9, 107.3, 108.9, 111.4, 118.6, 120.6, 120.7, 123.0, 124.1, 124.8, 126.6, 126.9, 127.7, 129.3, 129.4, 129.7, 134.4, 137.2, 139.8, 145.8, 147.9, 148.1, 155.9. HRMS-TOF: m/z $[M + H]^+$ 557.1843 (Calcd for $C_{30}H_{29}N_4O_5S$: 557.1853).

*6,7-dimethoxy-2-((4-(4-((o-tolyloxy)methyl)-1H-1,2,3-triazol-1-yl)phenyl)sulfonyl)-1,2,3,4-tetrahydroisoquinoline (**27**)*

White solid. mp 138-139 °C. IR (UATR) cm^{-1}: 1596, 1519, 1463, 1346, 1237, 1160. ^{1}H NMR (300 MHz, $CDCl_3$) δ 2.28 (s, 3H, CH_3), 2.86 (t, *J* = 5.9 Hz, 2H, C4-*H*), 3.44 (t, *J* = 5.9 Hz, 2H, C3-*H*), 3.84 (s, 6H, 2 × OCH_3), 4.28 (s, 2H, C1-*H*), 5.34 (s, 2H, CH_2O), 6.53 (s, 1H, Ar*H*), 6.57 (s, 1H, Ar*H*), 6.90-7.02 (m, 2H, Ar*H*), 7.16-7.23 (m, 2H, Ar*H*), 7.96 (d, *J* = 8.9 Hz, 2H, Ar*H*), 8.02 (d, *J* = 8.9 Hz, 2H, Ar*H*), 8.11 (s, 1H, C*H*N). ^{13}C NMR (75 MHz, $CDCl_3$) δ 16.3, 28.2, 43.8, 47.2, 55.9, 56.0, 62.1, 109.0, 111.5, 120.6, 121.3, 123.0, 124.2, 124.8, 127.0, 129.4, 129.5, 131.0, 137.2, 139.9, 146.3, 147.9, 148.1, 156.2. HRMS-TOF: m/z $[M + H]^+$ 521.1859 (Calcd for $C_{27}H_{29}N_4O_5S$: 521.1853).

*6,7-dimethoxy-2-((4-(4-((p-tolyloxy)methyl)-1H-1,2,3-triazol-1-yl)phenyl)sulfonyl)-1,2,3,4-tetrahydroisoquinoline (**28**)*

White solid. mp 168-169 °C. IR (UATR) cm^{-1}: 1596, 1509, 1464, 1346, 1225, 1162. ^{1}H NMR (300 MHz, $CDCl_3$) δ 2.31 (s, 3H, CH_3), 2.86 (t, *J* = 5.7 Hz, 2H, C4-*H*), 3.44 (t, *J* = 5.7 Hz, 2H, C3-*H*), 3.84 (s, 6H, 2 × OCH_3), 4.28 (s, 2H, C1-*H*), 5.30 (s, 2H, CH_2O), 6.53 (s, 1H, Ar*H*), 6.56 (s, 1H, Ar*H*), 6.92 (d, *J* = 8.5 Hz, 2H, Ar*H*), 7.12 (d, *J* = 8.5 Hz, 2H, Ar*H*), 7.94 (d, *J* = 8.7 Hz, 2H, Ar*H*), 8.01 (d, *J* = 8.7 Hz, 2H, Ar*H*), 8.13 (s, 1H, C*H*N). ^{13}C NMR (75 MHz, $CDCl_3$) δ 20.5, 28.2, 43.8, 47.2, 55.9, 56.0, 62.0, 109.0,

111.4, 114.6, 120.6, 123.0, 129.4, 130.1, 130.9, 137.2, 139.9, 146.1, 147.9, 148.1, 155.9. HRMS-TOF: m/z $[M + H]^+$ 521.1842 (Calcd for $C_{27}H_{29}N_4O_5S$: 521.1853).

*methyl 2-((1-(4-((6,7-dimethoxy-3,4-dihydroisoquinolin-2(1H)-yl)sulfonyl)phenyl)-1H-1,2,3-triazol-4-yl)methoxy)benzoate (**29**)*

White solid. mp 142-143 °C. IR (UATR) cm^{-1}: 1723, 1598, 1519, 1451, 1347, 1258, 1162. ^1H NMR (300 MHz, $CDCl_3$) δ 2.83 (t, J = 5.8 Hz, 2H, C4-*H*), 3.40 (t, J = 5.8 Hz, 2H, C3-*H*), 3.80 (s, 6H, 2 × OCH_3), 3.87 (s, 3H, CO_2CH_3), 4.24 (s, 2H, C1-*H*), 5.38 (s, 2H, CH_2O), 6.50 (s, 1H, Ar*H*), 6.53 (s, 1H, Ar*H*), 7.02 (t, J = 8.2 Hz, 1H, Ar*H*), 7.12 (d, J = 8.3 Hz, 1H, Ar*H*), 7.48 (dt, J = 8.2, 1.8 Hz, 1H, Ar*H*), 7.82 (dd, J = 7.8, 1.7 Hz, 1H, Ar*H*), 7.94 (d, J = 8.9 Hz, 2H, Ar*H*), 7.99 (d, J = 8.9 Hz, 2H, Ar*H*), 8.31 (s, 1H, C*H*N). ^{13}C NMR (75 MHz, $CDCl_3$) δ 28.3, 43.8, 47.2, 52.0, 55.9, 56.0, 63.4, 108.9, 111.4, 114.0, 120.5, 120.6, 121.0, 121.3, 123.0, 124.8, 129.4, 131.9, 133.8, 137.0, 139.9, 146.0, 147.8, 148.0, 157.8, 166.1. HRMS-TOF: m/z $[M + H]^+$ 565.1737 (Calcd for $C_{28}H_{29}N_4O_7S$: 565.1752).

*4-((1-(4-((6,7-dimethoxy-3,4-dihydroisoquinolin-2(1H)-yl)sulfonyl)phenyl)-1H-1,2,3-triazol-4-yl)methoxy)benzaldehyde (**30**)*

White solid. mp 186-187 °C. IR (UATR) cm^{-1}: 1692, 1604, 1519, 1348, 1231, 1162. ^1H NMR (300 MHz, $CDCl_3$) δ 2.86 (t, J = 5.8 Hz, 2H, C4-*H*), 3.45 (t, J = 5.8 Hz, 2H, C3-*H*), 3.84 (s, 6H, 2 × OCH_3), 4.29 (s, 2H, C1-*H*), 5.42 (s, 2H, CH_2O), 6.53 (s, 1H, Ar*H*), 6.56 (s, 1H, Ar*H*), 7.15 (d, J = 8.7 Hz, 2H, Ar*H*), 7.88 (d, J = 8.7 Hz, 2H, Ar*H*), 7.95 (d, J = 8.8 Hz, 2H, Ar*H*), 8.04 (d, J = 8.8 Hz, 2H, Ar*H*), 8.17 (s, 1H, C*H*N), 9.92 (s, 1H, C*H*O). ^{13}C NMR (75 MHz, $CDCl_3$) δ 28.2, 43.8, 47.2, 55.9, 56.0, 62.0, 109.0, 111.5, 115.1, 120.6, 120.9, 123.0, 124.8, 129.5, 130.6, 132.1, 137.5, 139.7, 144.8, 147.9, 148.1, 162.9, 190.6. HRMS-TOF: m/z $[M + H]^+$ 535.1645 (Calcd for $C_{27}H_{27}N_4O_6S$: 535.1646).

*3-((1-(4-((6,7-dimethoxy-3,4-dihydroisoquinolin-2(1H)-yl)sulfonyl)phenyl)-1H-1,2,3-triazol-4-yl)methoxy)-4-methoxybenzaldehyde (**31**)*

Pale yellow solid. mp 141-142 °C. IR (UATR) cm^{-1}: 1682, 1587, 1519, 1464, 1346, 1260, 1161. ^1H NMR (300 MHz, $CDCl_3$) δ 2.86 (t, J = 5.8 Hz, 2H, C4-*H*), 3.44 (t, J = 5.8 Hz, 2H, C3-*H*), 3.83, 3.84, 3.95 (3s, 9H, 3 × OCH_3), 4.27 (s, 2H, C1-*H*), 5.48 (s, 2H, CH_2O), 6.53 (s, 1H, Ar*H*), 6.56 (s, 1H, Ar*H*), 7.24 (d, J = 7.9 Hz, 1H, Ar*H*), 7.46 (s, 1H, Ar*H*), 7.47 (d, J = 8.0 Hz, 1H, Ar*H*), 7.94 (d, J = 8.8 Hz, 2H, Ar*H*), 8.01 (d, J = 8.8 Hz, 2H, Ar*H*), 8.21 (s, 1H, C*H*N), 9.88 (s, 1H, C*H*O). ^{13}C NMR (75 MHz, $CDCl_3$) δ 28.2, 43.8, 47.2, 55.9, 56.0, 62.7, 109.0, 109.5, 111.5, 112.6, 120.6, 121.2, 123.0, 124.8, 126.6, 129.4, 130.9, 137.4, 139.7, 144.8, 147.9, 148.1, 150.0, 152.8, 190.8. HRMS-TOF: m/z $[M + H]^+$ 565.1759 (Calcd for $C_{28}H_{29}N_4O_7S$: 565.1752).

*4-((1-(4-((6,7-dimethoxy-3,4-dihydroisoquinolin-2(1H)-yl)sulfonyl)phenyl)-1H-1,2,3-triazol-4-yl)methoxy)-3-methoxybenzaldehyde (**32**)*

Pale yellow solid. mp 116-117 °C. IR (UATR) cm^{-1}: 1683, 1596, 1518, 1437, 1346, 1264, 1162. ^{1}H NMR (300 MHz, $CDCl_3$) δ 2.87 (t, J = 5.7 Hz, 2H, C4-*H*), 3.46 (t, J = 5.9 Hz, 2H, C3-*H*), 3.84, 3.99 (2s, 9H, 3 × OCH_3), 4.28 (s, 2H, C1-*H*), 5.44 (s, 2H, CH_2O), 6.54 (s, 1H, Ar*H*), 6.57 (s, 1H, Ar*H*), 7.04 (d, J = 8.3 Hz, 1H, Ar*H*), 7.55 (dd, J = 8.2, 1.8 Hz, 1H, Ar*H*), 7.61 (d, J = 1.8 Hz, 1H, Ar*H*), 7.95 (d, J = 8.8 Hz, 2H, Ar*H*), 8.02 (d, J = 8.8 Hz, 2H, Ar*H*), 8.20 (s, 1H, C*H*N), 9.88 (s, 1H, C*H*O). ^{13}C NMR (75 MHz, $CDCl_3$) δ 28.2, 43.8, 47.2, 56.0, 56.2, 62.8, 108.9, 111.1, 111.4, 112.0, 120.7, 121.0, 123.0, 124.8, 127.2, 129.4, 130.1, 137.3, 139.8, 144.9, 148.1, 155.0, 190.6. HRMS-TOF: m/z $[M + H]^+$ 565.1753 (Calcd for $C_{28}H_{29}N_4O_7S$: 565.1752).
