# Supplementary material for: Discovery of novel 1,2,3-triazole derivatives as anticancer agents using QSAR and in silico structural modification
Source: Springerplus. 2015 Oct 5;4:571. doi: 10.1186/s40064-015-1352-5 (PMC4628044; doi:10.1186/s40064-015-1352-5)
Supplement: Supplementary file 2 — 10.1186/s40064-015-1352-5 Values of informative molecular descriptors of tested (1-32) and virtually modified compounds (1A-1R, 2A-2R, 7A-7R and 8A-8R). [file 40064_2015_1352_MOESM2_ESM.pdf]

Original Article: *SpringerPlus*

Discovery of novel 1,2,3-triazole derivatives as anticancer agents using QSAR and *in silico* structural modification

Veda Prachayasittikul[1,2], Ratchanok Pingaew[3], Nuttapat Anuwongcharoen[1,2] , Apilak Worachartcheewan[2,4], Chanin Nantasenamat[2], Supaluk Prachayasittikul[2]*, Somsak Ruchirawat[5,6,7]
Virapong Prachayasittikul[1]*

[1]*Department of Clinical Microbiology and Applied Technology, Faculty of Medical Technology, Mahidol University, Bangkok 10700, Thailand*

[2]*Center of Data Mining and Biomedical Informatics, Faculty of Medical Technology, Mahidol University, Bangkok 10700, Thailand*

[3]*Department of Chemistry, Faculty of Science, Srinakharinwirot University, Bangkok 10110, Thailand*

[4]*Department of Clinical Chemistry, Faculty of Medical Technology, Mahidol University, Bangkok 10700, Thailand*

[5]*Laboratory of Medicinal Chemistry, Chulabhorn Research Institute, Bangkok 10210, Thailand*

[6]*Program in Chemical Biology, Chulabhorn Graduate Institute, Bangkok 10210, Thailand*

[7]*Center of Excellence on Environmental Health and Toxicology, Commission on Higher Education (CHE), Ministry of Education, Thailand*

---

*Corresponding authors:

E-mail: virapong.pra@mahidol.ac.th; Telephone: 66-2-441-4376, Fax: 66-2-441-4380

E-mail: supaluk@swu.ac.th; Telephone: 66-2-441-4376, Fax: 66-2-441-4380

Table S1 Values of informative molecular descriptors of tested (**1-32**) and virtually modified compounds (**1A-1R**, **2A-2R**, **7A-7R** and **8A-8R**)

Compound	R5e+	nArCOOR	RDF105m	MATS7m	MATS8v	Lop	R7m
**1**	0.022	0	5.893	-0.045	0.258	0.434	0.318
**2**	0.029	0	6.774	-0.083	0.122	0.415	0.347
**3**	0.023	0	6.85	-0.062	0.068	0.383	0.333
**4**	0.026	0	7.661	-0.077	0.107	0.476	0.302
**5**	0.032	0	4.469	-0.088	0.056	0.620	0.331
**6**	0.028	0	9.058	-0.107	0.043	0.662	0.328
**7**	0.024	0	7.070	-0.082	0.130	0.476	0.352
**8**	0.021	1	6.311	-0.087	0.311	0.797	0.383
**9**	0.023	0	8.770	-0.077	-0.012	0.740	0.347
**10**	0.023	0	8.246	-0.081	0.023	0.740	0.368
**11**	0.027	0	6.394	-0.082	0.161	0.442	0.327
**12**	0.025	0	6.726	-0.081	0.276	0.442	0.369
**13**	0.034	0	9.540	0.062	0.340	0.767	0.297
**14**	0.032	0	6.382	0.012	0.156	0.825	0.336
**15**	0.033	0	8.157	-0.010	0.123	0.848	0.343
**16**	0.028	0	5.046	-0.034	0.143	0.721	0.314
**17**	0.028	0	7.637	-0.013	0.157	0.406	0.275
**18**	0.024	0	7.789	-0.001	0.096	0.375	0.284
**19**	0.022	0	6.934	-0.016	0.164	0.467	0.340
**20**	0.030	0	9.444	-0.017	0.249	0.651	0.362
**21**	0.023	1	12.250	-0.020	0.348	0.783	0.331
**22**	0.024	0	7.787	-0.018	0.082	0.608	0.289
**23**	0.024	0	4.972	-0.017	0.311	0.435	0.340
**24**	0.034	0	2.317	0.021	0.158	0.927	0.345
**25**	0.030	0	11.327	0.057	0.200	0.727	0.331
**26**	0.031	0	8.133	0.079	0.164	0.681	0.323

Table S1 Values of informative molecular descriptors of tested (**1-32**) and virtually modified compounds (**1A-1R**, **2A-2R**, **7A-7R** and **8A-8R**) (continue)

Compound	R5e+	nArCOOR	RDF105m	MATS7m	MATS8v	Lop	R7m
**27**	0.030	0	13.497	0.056	0.210	0.759	0.361
**28**	0.028	0	14.609	0.064	0.191	0.759	0.316
**29**	0.028	1	12.275	0.040	0.299	0.957	0.380
**30**	0.029	0	7.582	0.050	0.135	0.812	0.326
**31**	0.032	0	10.152	0.043	0.086	0.876	0.377
**32**	0.030	0	13.383	0.053	0.068	0.876	0.385
**1A**	0.031	0	6.506	-0.079	0.473	0.718	0.385
**1B**	0.038	0	9.271	-0.008	0.438	0.889	0.351
**1C**	0.029	0	7.551	-0.018	0.433	0.757	0.403
**1D**	0.034	0	6.424	-0.007	0.533	0.744	0.322
**1E**	0.027	0	7.230	-0.007	0.519	0.706	0.326
**1F**	0.035	0	10.675	0.024	0.412	0.877	0.367
**1G**	0.037	0	7.124	0.067	0.452	0.747	0.403
**1H**	0.027	0	7.072	0.024	0.533	0.694	0.342
**1J**	0.026	0	13.630	-0.081	0.375	0.718	0.390
**1K**	0.031	0	17.707	-0.011	0.373	0.889	0.368
**1L**	0.029	0	13.084	-0.016	0.375	0.757	0.411
**1M**	0.047	0	11.396	-0.007	0.397	0.744	0.337
**1N**	0.030	0	18.166	-0.008	0.385	0.706	0.380
**1P**	0.029	0	18.976	0.022	0.328	0.877	0.408
**1Q**	0.033	0	14.284	0.066	0.368	0.747	0.433
**1R**	0.038	0	13.964	0.013	0.367	0.694	0.324
**2A**	0.032	0	7.875	-0.123	-0.020	0.694	0.355
**2B**	0.030	0	12.834	-0.047	0.054	0.865	0.361
**2C**	0.028	0	10.894	-0.055	0.054	0.736	0.372
**2D**	0.028	0	5.770	-0.049	-0.038	0.718	0.314
**2E**	0.027	0	9.722	-0.047	-0.005	0.683	0.320

Table S1 Values of informative molecular descriptors of tested (**1-32**) and virtually modified compounds (**1A-1R**, **2A-2R**, **7A-7R** and **8A-8R**) (continue)

Compound	R5e+	nArCOOR	RDF105m	MATS7m	MATS8v	Lop	R7m
**2F**	0.029	0	11.196	-0.012	0.034	0.853	0.390
**2G**	0.035	0	11.933	0.035	0.060	0.726	0.370
**2H**	0.026	0	7.484	-0.015	-0.008	0.672	0.297
**2J**	0.030	0	9.136	-0.124	-0.183	0.694	0.359
**2K**	0.028	0	13.039	-0.048	-0.070	0.865	0.345
**2L**	0.029	0	12.228	-0.053	-0.068	0.736	0.369
**2M**	0.050	0	4.187	-0.046	-0.216	0.718	0.323
**2N**	0.029	0	17.392	-0.045	-0.174	0.683	0.350
**2P**	0.029	0	12.316	-0.011	-0.088	0.853	0.425
**2Q**	0.027	0	11.144	0.037	-0.065	0.726	0.378
**2R**	0.025	0	5.369	-0.023	-0.177	0.672	0.269
**7A**	0.048	0	12.501	-0.121	0.153	0.848	0.419
**7B**	0.049	0	15.644	-0.051	0.174	0.997	0.393
**7C**	0.052	0	12.966	-0.058	0.167	0.875	0.439
**7D**	0.057	0	8.593	-0.052	0.149	0.876	0.339
**7E**	0.046	0	12.039	-0.051	0.177	0.835	0.406
**7F**	0.045	0	21.536	-0.019	0.156	0.984	0.433
**7G**	0.046	0	13.458	0.026	0.177	0.864	0.473
**7H**	0.047	0	10.492	-0.020	0.182	0.822	0.283
**7J**	0.046	0	13.358	-0.122	0.024	0.848	0.423
**7K**	0.041	0	19.251	-0.052	0.072	0.997	0.375
**7L**	0.047	0	15.847	-0.056	0.068	0.875	0.412
**7M**	0.056	0	4.830	-0.049	0.003	0.876	0.337
**7N**	0.037	0	7.848	-0.048	0.036	0.835	0.411
**7P**	0.035	0	8.466	-0.017	0.051	0.984	0.421
**7Q**	0.050	0	11.679	0.028	0.070	0.864	0.431
**7R**	0.051	0	18.007	-0.027	0.032	0.822	0.360

Table S1 Values of informative molecular descriptors of tested (**1-32**) and virtually modified compounds (**1A-1R**, **2A-2R**, **7A-7R** and **8A-8R**) (continue)

Compound	R5e+	nArCOOR	RDF105m	MATS7m	MATS8v	Lop	R7m
**8A**	0.030	1	8.078	-0.160	0.116	0.973	0.457
**8B**	0.032	1	11.267	-0.096	0.146	1.113	0.443
**8C**	0.036	1	13.156	-0.105	0.144	0.997	0.487
**8D**	0.052	1	10.755	-0.100	0.109	1.005	0.365
**8E**	0.024	1	7.277	-0.092	0.135	0.958	0.457
**8F**	0.032	1	13.768	-0.063	0.125	1.098	0.482
**8G**	0.032	1	11.498	-0.023	0.150	0.983	0.519
**8H**	0.031	1	19.132	-0.062	0.136	0.943	0.418
**8J**	0.035	1	9.991	-0.158	-0.006	0.973	0.464
**8K**	0.036	1	14.044	-0.094	0.049	1.113	0.413
**8L**	0.027	1	11.557	-0.099	0.049	0.997	0.455
**8M**	0.030	1	8.474	-0.093	-0.027	1.005	0.397
**8N**	0.022	1	6.857	-0.085	0.004	0.958	0.455
**8P**	0.031	1	11.989	-0.058	0.027	1.098	0.489
**8Q**	0.035	1	7.017	-0.016	0.050	0.983	0.537
**8R**	0.026	1	13.660	-0.064	-0.001	0.943	0.388
