# Supplementary material for: Discovery of novel 1,2,3-triazole derivatives as anticancer agents using QSAR and in silico structural modification
Source: Springerplus. 2015 Oct 5;4:571. doi: 10.1186/s40064-015-1352-5 (PMC4628044; doi:10.1186/s40064-015-1352-5)
Supplement: Supplementary file 3 — 10.1186/s40064-015-1352-5 Predicted cytotoxic activity (pIC50) of modified compounds (1A-1R, 2A-2R, 7A-7R and 8A-8R) and experimental cytotoxic activity of reference drugs. [file 40064_2015_1352_MOESM3_ESM.pdf]

Original Article: *SpringerPlus*

Discovery of novel 1,2,3-triazole derivatives as anticancer agents using QSAR and *in silico* structural modification

Veda Prachayasittikul[1,2], Ratchanok Pingaew[3], Nuttapat Anuwongcharoen[1,2] , Apilak Worachartcheewan[2,4], Chanin Nantasenamat[2], Supaluk Prachayasittikul[2]*, Somsak Ruchirawat[5,6,7]
Virapong Prachayasittikul[1]*

[1]*Department of Clinical Microbiology and Applied Technology, Faculty of Medical Technology, Mahidol University, Bangkok 10700, Thailand*

[2]*Center of Data Mining and Biomedical Informatics, Faculty of Medical Technology, Mahidol University, Bangkok 10700, Thailand*

[3]*Department of Chemistry, Faculty of Science, Srinakharinwirot University, Bangkok 10110, Thailand*

[4]*Department of Clinical Chemistry, Faculty of Medical Technology, Mahidol University, Bangkok 10700, Thailand*

[5]*Laboratory of Medicinal Chemistry, Chulabhorn Research Institute, Bangkok 10210, Thailand*

[6]*Program in Chemical Biology, Chulabhorn Graduate Institute, Bangkok 10210, Thailand*

[7]*Center of Excellence on Environmental Health and Toxicology, Commission on Higher Education (CHE), Ministry of Education, Thailand*

---

*Corresponding authors:

E-mail: virapong.pra@mahidol.ac.th; Telephone: 66-2-441-4376, Fax: 66-2-441-4380

E-mail: supaluk@swu.ac.th; Telephone: 66-2-441-4376, Fax: 66-2-441-4380

Table S2 Predicted cytotoxic activity (pIC_{50}) of modified compounds (**1A-1R**, **2A-2R**, **7A-7R** and **8A-8R)** and experimental cytotoxic activity of reference drugs

Compound	pIC_{50}			
	**HuCCA-1**	**HepG2**	**A549**	**MOLT-3**
**1A**	-1.731[c]	-1.652[c]	-1.027[c]	-0.915[b]
**1B**	-2.319[c]	-1.067[c]	-1.083[c]	-1.087[c]
**1C**	-1.563[c]	-1.254[c]	-1.091[c]	-0.687[b]
**1D**	-1.983[c]	-1.285[c]	-0.931[b]	-1.543[c]
**1E**	-1.395[c]	-1.222[c]	-0.954[b]	-1.542[c]
**1F**	-2.067[c]	-0.791[b]	-1.125[c]	-0.933[b]
**1G**	-2.235[c]	-0.846 [b]	-1.061[c]	-0.697[b]
**1H**	-1.395[c]	-1.073[c]	-0.931[b]	-1.388[c]
**1J**	-1.311[c]	-1.104[c]	-1.184[c]	-0.864[b]
**1K**	-1.731[c]	-0.421[b]	-1.187[c]	-0.910[b]
**1L**	-1.563[c]	-0.809[b]	-1.184[c]	-0.604[b]
**1M**	-3.075[c]	-0.895[b]	-1.149[c]	-1.387[c]
**1N**	-1.647[c]	-0.369[b]	-1.168[c]	-0.980[b]
**1P**	-1.563[c]	-0.151[b,d]	-1.259[c]	-0.507[b]
**1Q**	-1.898[c]	-0.290[b]	-1.195[c]	-0.386[b]
**1R**	-2.319[c]	-0.590[b]	-1.196[c]	-1.575[c]
**2A**	-1.815[c]	-1.773[c]	-1.815[c]	-1.253[c]
**2B**	-1.647[c]	-0.990[b]	-1.697[c]	-1.008[c]
**2C**	-1.479[c]	-1.184[c]	-1.697[c]	-1.031[c]
**2D**	-1.479[c]	-1.554[c]	-1.844[c]	-1.654[c]
**2E**	-1.395[c]	-1.234[c]	-1.791[c]	-1.629[c]
**2F**	-1.563[c]	-0.937[b]	-1.729[c]	-0.720[b]
**2G**	-2.067[c]	-0.635[b]	-1.687[c]	-1.063[c]
**2H**	-1.311[c]	-1.243[c]	-1.796[c]	-1.879[c]
**2J**	-1.647[c]	-1.679[c]	-2.075[c]	-1.211[c]
**2K**	-1.479[c]	-0.979[b]	-1.895[c]	-1.175[c]
**2L**	-1.563[c]	-1.069[c]	-1.892[c]	-1.063[c]
**2M**	-3.327[c]	-1.663[c]	-2.128[c]	-1.560[c]

[a] Highly active compound. [b] Moderately active compound.[c] Weakly active to inactive compound. [d] The most potent compound against each cancer cell line. [e] Not tested. [f] Experimental pIC_{50} values.

Table S2 Predicted cytotoxic activity (pIC_{50}) of modified compounds (**1A-1R**, **2A-2R**, **7A-7R** and **8A-8R)** and experimental cytotoxic activity of reference drugs (continue)

Compound	pIC_{50}			
	**HuCCA-1**	**HepG2**	**A549**	**MOLT-3**
**2N**	-1.563[c]	-0.622[b]	-2.061[c]	-1.317[c]
**2P**	-1.563[c]	-0.844[b]	-1.924[c]	-0.356[b]
**2Q**	-1.395[c]	-0.687[b]	-1.887[c]	-0.980[b]
**2R**	-1.227[c]	-1.451[c]	-2.066[c]	-2.171[c]
**7A**	-3.159[c]	-1.400[c]	-1.538[c]	-0.424[b]
**7B**	-3.243[c]	-0.790[b]	-1.505[c]	-0.535[b]
**7C**	-3.495[c]	-1.037[c]	-1.516[c]	-0.187[b]
**7D**	-3.915[c]	-1.348[c]	-1.545[c]	-1.226[c]
**7E**	-2.991[c]	-1.073[c]	-1.500[c]	-0.573[b]
**7F**	-2.907[c]	-0.163[b]	-1.534[c]	-0.133[b]
**7G**	-2.991[c]	-0.562[b]	-1.500[c]	0.155[a]
**7H**	-3.075[c]	-1.034[c]	-1.492[c]	-1.865[c]
**7J**	-2.991[c]	-1.338[c]	-1.745[c]	-0.382[b]
**7K**	-2.571[c]	-0.513[b]	-1.668[c]	-0.722[b]
**7L**	-3.075[c]	-0.801[b]	-1.674[c]	-0.468[b]
**7M**	-3.831[c]	-1.628[c]	-1.778[c]	-1.246[c]
**7N**	-2.235[c]	-1.386[c]	-1.725[c]	-0.521[b]
**7P**	-2.067[c]	-1.177[c]	-1.701[c]	-0.258[b]
**7Q**	-3.327[c]	-0.692[b]	-1.671[c]	-0.282[b]
**7R**	-3.411[c]	-0.481[b]	-1.732[c]	-1.065[c]
**8A**	-0.618[b]	-1.949[c]	-0.672[b]	0.105[a]
**8B**	-0.786[b]	-1.367[c]	-0.625[b]	0.108[a]
**8C**	-1.122[c]	-1.266[c]	-0.628[b]	0.442[a]
**8D**	-2.466[c]	-1.428[c]	-0.684[b]	-0.818[b]
**8E**	-0.114[b]	-1.659[c]	-0.642[b]	0.089[a]
**8F**	-0.786[b]	-0.999[b]	-0.658[b]	0.498[a]
**8G**	-0.786[b]	-0.970[b]	-0.618[b,d]	0.760[a]

[a] Highly active compound. [b] Moderately active compound.[c] Weakly active to inactive compound. [d] The most potent compound against each cancer cell line. [e] Not tested. [f] Experimental pIC_{50} values.

Table S2 Predicted cytotoxic activity (pIC_{50}) of modified compounds (**1A-1R**, **2A-2R**, **7A-7R** and **8A-8R)** and experimental cytotoxic activity of reference drugs (continue)

Compound	pIC_{50}			
	**HuCCA-1**	**HepG2**	**A549**	**MOLT-3**
**8H**	-0.702[b]	-0.574[b]	-0.640[b]	-0.333[b]
**8J**	-1.038[c]	-1.789[c]	-0.867[b]	0.177[a]
**8K**	-1.122[c]	-1.139[c]	-0.779[b]	-0.204[b]
**8L**	-0.366[b]	-1.391[c]	-0.779[b]	0.110[a]
**8M**	-0.618[b]	-1.571[c]	-0.901[b]	-0.485[b]
**8N**	0.054[a,d]	-1.656[c]	-0.851[b]	0.068[a]
**8P**	-0.702[b]	-1.113[c]	-0.815[b]	0.571[a]
**8Q**	-1.038[c]	-1.285[c]	-0.778[b]	0.947[a,d]
**8R**	-0.282[b]	-1.013[c]	-0.859[b]	-0.645[b]
Etoposide	-[e]	-1.479[f]	-[e]	1.292[f]
Doxorubicin	0.081[f]	0.102[f]	0.357[f]	-[e]

[a] Highly active compound. [b] Moderately active compound. [c] Weakly active to inactive compound. [d] The most potent compound against each cancer cell line. [e] Not tested. [f] Experimental pIC_{50} values.
