# Supplementary material for: Discovery of novel 1,2,3-triazole derivatives as anticancer agents using QSAR and in silico structural modification
Source: Springerplus. 2015 Oct 5;4:571. doi: 10.1186/s40064-015-1352-5 (PMC4628044; doi:10.1186/s40064-015-1352-5)
Supplement: Supplementary file 4 — 10.1186/s40064-015-1352-5 A summary of substituent effects on cytotoxic activity of four cancer cell lines. [file 40064_2015_1352_MOESM4_ESM.pdf]

Original Article: *SpringerPlus*

Discovery of novel 1,2,3-triazole derivatives as anticancer agents using QSAR and *in silico* structural modification

Veda Prachayasittikul[1,2], Ratchanok Pingaew[3], Nuttapat Anuwongcharoen[1,2] , Apilak Worachartcheewan[2,4], Chanin Nantasenamat[2], Supaluk Prachayasittikul[2]*, Somsak Ruchirawat[5,6,7]
Virapong Prachayasittikul[1]*

[1]*Department of Clinical Microbiology and Applied Technology, Faculty of Medical Technology, Mahidol University, Bangkok 10700, Thailand*

[2]*Center of Data Mining and Biomedical Informatics, Faculty of Medical Technology, Mahidol University, Bangkok 10700, Thailand*

[3]*Department of Chemistry, Faculty of Science, Srinakharinwirot University, Bangkok 10110, Thailand*

[4]*Department of Clinical Chemistry, Faculty of Medical Technology, Mahidol University, Bangkok 10700, Thailand*

[5]*Laboratory of Medicinal Chemistry, Chulabhorn Research Institute, Bangkok 10210, Thailand*

[6]*Program in Chemical Biology, Chulabhorn Graduate Institute, Bangkok 10210, Thailand*

[7]*Center of Excellence on Environmental Health and Toxicology, Commission on Higher Education (CHE), Ministry of Education, Thailand*

---

*Corresponding authors:

E-mail: virapong.pra@mahidol.ac.th; Telephone: 66-2-441-4376, Fax: 66-2-441-4380

E-mail: supaluk@swu.ac.th; Telephone: 66-2-441-4376, Fax: 66-2-441-4380

Table S3 A summary of substituent effects on cytotoxic activity of four cancer cell lines

	HuCCA-1	HepG2	A549	MOLT-3
**Methoxy** 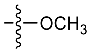	↑activity: 27 > 19, 30 > 22	↑activity: 9 >5, 24 >16, 27 > 19, 25 > 17, 26 > 18, 29 > 21	↑activity: 13 >1, 9 >5, 14 >5, 24 >16, 27 > 19, 30 >22	↑activity: 13>1, 9 >5, 14 >5, 15 >6, 26 >18, 29 > 21, 30 >22, 32 >30
	↓activity: 1 > 13, 5 > 14, 21 > 29, 16 >24, 30>32, 5>9	↓activity: 1 > 13, 2 > 14, 22 > 30, 30 >32	↓activity: 21 > 29, 30 > 32	
**Naphthalenyl & coumarinyl**				
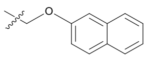 naphthalenyl	coumarin : ↑activity	coumarin : ↑ activity	coumarin : ↑ activity	coumarin : ↓activity
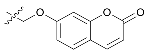 7-courmarinyl	coumarin (11) > naphthalene (3)	coumarin (11) > naphthalene (3)	coumarin (11) > naphthalene (3)	naphthalene (3) > coumarin (11)
	opened chain: 7-coumarin (11) > 4-coumarin (12)	opened chain: 7-coumarin (11) > 4-coumarin (12)	opened chain: 7-coumarin (11) > 4-coumarin (12)	opened chain: 4-coumarin (12) > 7-coumarin (11)
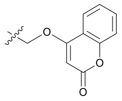 4-courmarinyl	For 4-coumarin : closed (23) > opened (12)	For 4-coumarin : no effect of closed/opened chain (23 ≈ 12)	For 4-coumarin : closed (23) > opened (12)	For 4-coumarin : opened (12) > closed (23)
**Methyl and acetyl**				
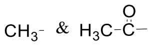 (19) (20)	↑ activity: 20 > 19	↑ activity: 20 > 19	↑ activity: 20 > 19	↑ activity: 20 > 19
**Position of methyl substituent on phenoxy ring**				
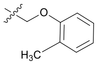 *ortho* 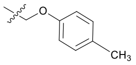 *para*	*ortho* > *para* : 27 > 28	*para* > *ortho* : 28 > 27	*ortho* > *para* : 27 > 28	*ortho* ≈ *para* : 27 ≈ 28

Table S3 A summary of substituent effects on cytotoxic activity of four cancer cell lines (continue)

	**HuCCA-1**	**HepG2**	**A549**	**MOLT-3**
**Relative position of -CHO and –OCH_3 substituents to each other**				
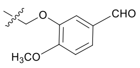<br>*para*<br>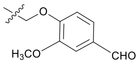<br>*meta*	*meta* > *para* :<br>32 > 31	*para* > *meta* :<br>31 > 32	*para* > *meta* :<br>10 > 9<br>31 > 32	*meta* > *para* :<br>32 > 31
**Adamantyl & phenyl of series 1, 2, 7 and 8**				
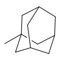<br>Adamantyl<br>(1-Adm)<br>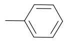<br>Phenyl<br>(C_6H_5)	Both *meta*- and *para*-triazole:<br>adamantyl > phenyl<br><br>(1H > 1D, 1R >1M, 2H > 2D, 2R >2M, 7H > 7D, 7R >7M, 8H > 8D, 8R > 8M)	Both *meta*- and *para*-triazole:<br>adamantyl > phenyl<br><br>(1H > 1D, 1R >1M, 2H > 2D, 2R >2M, 7H > 7D, 7R >7M, 8H > 8D, 8R > 8M)	Both *meta*- and *para*-triazole:<br>adamantyl > phenyl<br><br>(2H > 2D, 2R >2M, 7H > 7D, 7R >7M, 8H > 8D, 8R > 8M)<br><br>Except:<br>1H = 1D and<br>1M > 1R	*para*-triazole:<br>series **2** and **7**:<br>phenyl > adamantyl<br><br>(2D >2H, 7D >7H)<br>series **1** and **8**:<br>adamantyl > phenyl<br>(1H >1D, 8H >8D)<br><br>*meta*-triazole:<br>series **1**, **2**, **8**:<br>phenyl > adamantly<br>(1M > 1R, 2M >2R, 8M >8R)<br>series **7**:<br>adamantyl > phenyl<br>(7R >7M)
**Opened & closed chain** (focus on the series with most improved activity)				
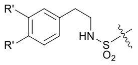<br>Opened<br>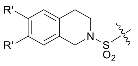<br>Closed	Both *meta*- and *para*-triazole:<br>Closed > opened<br><br>(1E >1A, 1F >1B, 1P >1K, 2E >2A, 2F >2B, 2N >2J, 2Q >2L, 7E >7A, 7F >7B, 7G >7C, 7N >7J, 7P >7K, 8E >8A, 8G >8C, 8N > 8J, 8P >8K)<br><br>Except :<br>1C >1G, 1J > 1N, 1L > 1Q, 2C >2G, 2K > 2P, 7L > 7Q, 8F = 8B, 8L > 8Q	Both *meta*- and *para*-triazole:<br>Closed > opened<br><br>(1E >1A, 1F >1B, 1G >1C, 1N >1J, 1P >1K, 1Q >1L, 2E >2A, 2F >2B, 2G >2C, 2N >2J, 2P >2K, 2Q >2L, 7E >7A, 7F >7B, 7G >7C, 7Q >7L, 8E >8A, 8F >8B, 8G >8C, 8N >8J, 8P >8K, 8Q >8L)<br><br>Except :<br>7J >7N, 7K > 7P	Both *meta*- and *para*-triazole:<br>Closed > opened<br><br>(1E >1A, 1G >1C, 1N >1J, 2E >2A, 2G >2C, 2N >2J, 2Q >2L, 7E >7A, 7G >7C, 7N >7J, 7K >7P, 7Q >7L, 8E >8A, 8G >8C, 8N >8J)<br><br>Except :<br>1B>1F, 1K >1P, 1L > 1Q, 2B>2F, 2K >2P, 7B>7F, 8B>8F, 8K>8P and 8Q ≈ 8L	Both *meta*- and *para*-triazole:<br>Closed > opened<br><br>(1F >1B, 1P >1K, 1Q >1L, 2F >2B, 2P >2K, 2Q >2L, 7F >7B, 7G >7C, 7P >7K, 7Q >7L, 8F >8B, 8G>8C, 8P >8K, 8Q > 8L)<br><br>Except:<br>1A >1E, 1C >1G, 1J > 1N, 2A >2E, 2C >2G, 2J > 2N, 7A >7E, 7J > 7N, 8A >8E, 8J >8N
