# Supplementary material for: Discovery of novel 1,2,3-triazole derivatives as anticancer agents using QSAR and in silico structural modification
Source: Springerplus. 2015 Oct 5;4:571. doi: 10.1186/s40064-015-1352-5 (PMC4628044; doi:10.1186/s40064-015-1352-5)
Supplement: Supplementary file 5 — 10.1186/s40064-015-1352-5 Comparison of meta- and para-triazole derivatives focused on the modified compound series with the best improved activity. [file 40064_2015_1352_MOESM5_ESM.pdf]

Original Article: *SpringerPlus*

Discovery of novel 1,2,3-triazole derivatives as anticancer agents using QSAR and *in silico* structural modification

Veda Prachayasittikul[1,2], Ratchanok Pingaew[3], Nuttapat Anuwongcharoen[1,2] , Apilak Worachartcheewan[2,4], Chanin Nantasenamat[2], Supaluk Prachayasittikul[2]*, Somsak Ruchirawat[5,6,7]
Virapong Prachayasittikul[1]*

[1]*Department of Clinical Microbiology and Applied Technology, Faculty of Medical Technology, Mahidol University, Bangkok 10700, Thailand*

[2]*Center of Data Mining and Biomedical Informatics, Faculty of Medical Technology, Mahidol University, Bangkok 10700, Thailand*

[3]*Department of Chemistry, Faculty of Science, Srinakharinwirot University, Bangkok 10110, Thailand*

[4]*Department of Clinical Chemistry, Faculty of Medical Technology, Mahidol University, Bangkok 10700, Thailand*

[5]*Laboratory of Medicinal Chemistry, Chulabhorn Research Institute, Bangkok 10210, Thailand*

[6]*Program in Chemical Biology, Chulabhorn Graduate Institute, Bangkok 10210, Thailand*

[7]*Center of Excellence on Environmental Health and Toxicology, Commission on Higher Education (CHE), Ministry of Education, Thailand*

---

*Corresponding authors:

E-mail: virapong.pra@mahidol.ac.th; Telephone: 66-2-441-4376, Fax: 66-2-441-4380

E-mail: supaluk@swu.ac.th; Telephone: 66-2-441-4376, Fax: 66-2-441-4380

Table S4 Comparison of *meta*- and *para*-triazole derivatives focused on the modified compound series with the best improved activity (see Fig. 7)

**Cell line**<br>(modified series with the best improved activity)	**opened / closed chain**	**R, R^1**	**R, R^1**	**R, R^1**	**R, R^2**
HuCCA-1<br>(series **8**)	Opened	*para* > *meta*<br>(8A > 8J)<br>R^1 = H	*para* > *meta*<br>(8B > 8K)<br>$R^1 = OCH_3$	*meta* > *para*<br>(8L > 8C)<br>R^1 = OH	*meta* > > *para*<br>(8M >> 8D)<br>$R^2 = C_6H_5$
R = 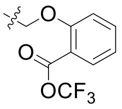	Closed	*meta* > > *para*<br>(8N >> 8E)<br>R^1 = H	*meta* > *para*<br>(8P > 8F)<br>$R^1 = OCH_3$	*para* > *meta*<br>(8G > 8Q)<br>R^1 = OH	*meta* > *para*<br>(8R > 8H)<br>R^2 = 1-Adm
HepG2<br>(series **1** and **7**)<br>Series **1**:<br>R = 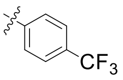	Opened	*meta* > *para*<br>(1J > 1A,<br>7J >7A)<br>R^1 = H	*meta* > *para*<br>(1K > 1B,<br>7K >7B)<br>$R^1 = OCH_3$	*meta* > *para*<br>(1L > 1C,<br>7L >7C)<br>R^1 = OH	*meta* > *para*<br>(1M>1D)<br>$R^2 = C_6H_5$<br>*para* > *meta*<br>(7D > 7M)<br>$R^2 = C_6H_5$
Series **7**:<br>R = 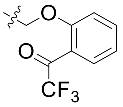	Closed	*meta* > *para*<br>(1N >1E)<br>*para* > *meta*<br>(7E > 7N)<br>R^1 = H	*meta* >> *para*<br>(1P >>1F)<br>*para* >> *meta*<br>(7F > 7P)<br>$R^1 = OCH_3$	*meta* > *para*<br>(1Q >1G)<br>*para* > *meta*<br>(7G > 7Q)<br>R^1 = OH	*meta* >> *para*<br>(1R >>1H,<br>7R >> 7H)<br>R^2 = 1-Adm
A549<br>(series **8**)	Opened	*para* > *meta*<br>(8A > 8J)<br>R^1 = H	*para* > *meta*<br>(8B > 8K)<br>$R^1 = OCH_3$	*para* > *meta*<br>(8C > 8L)<br>R^1 = OH	*para* > *meta*<br>(8D > 8M)<br>$R^2 = C_6H_5$
R = 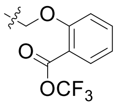	Closed	*para* > *meta*<br>(8E > 8N)<br>R^1 = H	*para* > *meta*<br>(8F > 8P)<br>$R^1 = OCH_3$	*para* > *meta*<br>(8G > 8Q)<br>R^1 = OH	*para* > *meta*<br>(8H > 8R)<br>R^2 = 1-Adm

Table S4 Comparison of *meta*- and *para*-triazole derivatives focused on the modified compound series with the best improved activity (continue)

**Cell line**<br>(modified series with the best improved activity)	**opened / closed chain**	**R, R^1**	**R, R^1**	**R, R^1**	**R, R^2**
**MOLT-3**<br>(series **8**)	Opened	*meta* > *para*<br>(8J >8A)<br>R1 = H	*para* > *meta*<br>(8B > 8K)<br>R1 = OCH3	*para* > *meta*<br>(8C > 8L)<br>R1 = OH	*meta* > *para*<br>(8M > 8D)<br>R2 = C6H5
R = 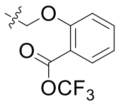	Closed	*para* > *meta*<br>(8E > 8N)<br>R1 = H	*meta* > *para*<br>(8P > 8F)<br>R1 = OCH3	*meta* > *para*<br>(8Q >8G)<br>R1 = OH	*para* > *meta*<br>(8H > 8R)<br>R2 = 1-Adm
